# Comparative Analysis of Mitochondrial Genomes among Twelve Sibling Species of the Genus *Atkinsoniella* Distant, 1908 (Hemiptera: Cicadellidae: Cicadellinae) and Phylogenetic Analysis

**DOI:** 10.3390/insects13030254

**Published:** 2022-03-03

**Authors:** Yan Jiang, Hao-Xi Li, Xiao-Fei Yu, Mao-Fa Yang

**Affiliations:** 1 Institute of Entomology, Guizhou University, Guiyang 550025, China; yanjianggz@outlook.com; 2 Guizhou Provincial Key Laboratory for Agricultural Pest Management of the Mountainous Region, Guiyang 550025, China; lhx_ppath@sina.com (H.-X.L.); anjingfly2009@163.com (X.-F.Y.); 3 College of Tobacco Sciences, Guizhou University, Guiyang 550025, China

**Keywords:** leafhopper, Cicadellinae, *Atkinsoniella*, mitogenome, phylogenetic analysis

## Abstract

**Simple Summary:**

*Atkinsoniella* is a large genus of 98 species across the world and 88 species recorded in China within the globally distributed subfamily Cicadellinae, which is phytophagous, and some of which have been reported as important agricultural pests. Some *Atkinsoniella* species are very similar in morphological characteristics, making accurate identification at species level confusing. To provide further evidence toward understanding the relationships within the genus *Atkinsoniella* and subfamily Cicadellinae, mitogenomes of 12 *Atkinsoniella* sibling species were obtained and annotated. Their characteristics were comparatively analyzed. In addition, the comprehensive phylogenetic relationship within the subfamily Cicadellinae was determined based on three mitochondrial datasets using both the maximum-likelihood (ML) and Bayesian inference (BI) methods. The results suggested that the genus *Atkinsoniella* was recovered as a monophyletic group. The branches of the 12 newly sequenced species were clearly separated, with most nodes receiving strong support in all analyses, indicating that mitogenomics is an effective method for identifying closely related species and understanding their phylogenetic and evolutionary relationships.

**Abstract:**

The herbivorous leafhopper genus *Atkinsoniella* Distant, 1908 (Hemiptera: Cicadellidae: Cicadellinae), a large genus of subfamily Cicadellinae, consists of 98 valid species worldwide and 88 species recorded in China. Some species of the genus are very similar in morphological characteristics, so they are difficult to identify accurately. In this study, 12 mitochondrial genomes of *Atkinsoniella* species with similar morphological characteristics were first obtained through high-throughput sequencing, which featured a typical circular molecule of 15,034–15,988 bp in length. The arrangement and orientation of 37 genes were identical to those of typical Cicadellidae mitogenomes. The phylogenetic relationship within the subfamily Cicadellinae was reconstructed using maximum-likelihood (ML) and Bayesian inference (BI) methods based on three concatenated datasets. The topological structures of the six obtained phylogenetic trees were highly consistent. The results suggested that *Atkinsoniella* was recovered as a monophyletic group and emerged as a sister group with the monophyletic clade of *Bothrogonia*, *Paracrocampsa* (part), and *Draeculacephala* (part). The branches of the 12 newly sequenced species were clearly separated, with most nodes receiving strong support in all analyses. In addition, the key to the 12 *Atkinsoniella* species was provided to identify species according to morphological characteristics. This study further promotes research on the classification, genetics, evolution, and phylogeny of the genus *Atkinsoniella* and subfamily Cicadellinae.

## 1. Introduction

Cicadellinae is a relatively large subfamily within the family Cicadellidae (Hemiptera: Auchenorrhyncha: Cicadelloidea), which is the largest family in Hemiptera, with over 23,000 species in 25 subfamilies [1,2]. Cicadellinae is one of the largest and most brightly colored groups of leafhoppers, is distributed throughout all of the zoogeographical realms of the world, and is especially rich in species from tropical and subtropical regions. There are approximately 2400 species in approximately 330 genera distributed worldwide, and 259 species in 23 genera have been recorded in China now that *Mileewa*, *Ujna*, and *Processina* have been placed into the subfamily Mileewinae [3,4,5,6,7]. According to our experience collecting Cicadellinae samples in the wild, they prefer to live in the humid and well-lit forests of the mountains. Some Cicadellinae species are of considerable economic importance because they feed on the sap in the xylem of woody and herbaceous plants and are important vectors of the xylem-limited bacterial pathogen *Xylella fastidiosa*, which induces diseases of grapevines, citrus, alfalfa, almond, coffee, stone fruits, and landscape ornamentals [8,9,10,11].

*Atkinsoniella*, a large genus within the subfamily Cicadellinae, is mainly distributed in the Oriental realm and the Palearctic realm, with almost 98 valid species distributed worldwide and 88 species recorded in China [3,7]. Currently, the identification of Cicadellinae insects is mainly based on the external morphological and male genitalia characteristics of adults. However, some adult Cicadellinae species exhibit sexual dimorphism and polymorphism, showing differences in body color, markings, and individual size between male and female individuals, and there are both long-winged and short-winged types in the same sex of one species [3]. *Atkinsoniella* exhibits extremely similar morphological characteristics among some species, such as the 12 species that are included in this study. Their wings are all yellow with relatively few variable markings. It is very difficult to accurately identify the species without male genitalia, especially for the specimens that have been soaked in ethanol. As important morphological characteristics for identifying Cicadellinae species, the male genitalia of 12 *Atkinsoniella* species showed little difference, causing some yellow-winged *Atkinsoniella* species samples to be identified only to the genus level. 

Therefore, the identification of yellow-winged species based on male genitalia is also confusing. Females can be identified as *Atkinsoniella* spp., making the identification of *Atkinsoniella* at the species level more challenging. Therefore, molecular methods are needed to validate and complement the morphological classification of some difficult-to-identify species. To date, only 41 molecular records of nine *Atkinsoniella* species (*A*. *alternata* Young, 1986; *A*. *dactylia* Yang *et* Li, 2000; *A*. *grahami* Young, 1986; *A*. *heiyuana* Li, 1992; *A*. *opponens* (Walker, 1851); *A*. *sulphurate* (Distant, 1908); *A*. *thalia* (Distant, 1918); *A*. *trimaculata* Li, 1992; *A*. *xanthonota* Kuoh, 1994) can be retrieved from the NCBI database, but most of them are molecular fragments (*28S*, *COX1*, *COX2*, *H3*, *16S*, *CYTB*, *SSU*), and only three of them are complete mitochondrial genome (mitogenome) sequences: *A*. *grahami*, MW533712; *A*. *xanthonota*, MW533713, MT596736. Insect mitogenomes are usually double-stranded circular molecules with a typical length of 14.5–17.0 kb, encoding 13 protein-coding genes (PCGs), 22 transfer RNAs (tRNAs), two ribosomal RNAs (rRNAs), and a control region (CR) that contains the initial sites for replication and transcription [12,13,14]. The materially inherited mitogenome is an informative molecular marker for species identification [15,16], population genetics [17,18,19], evolutionary genomics, and phylogenetic analysis of various taxonomic levels [13,20,21,22,23,24,25] due to its highly conserved order and orientation, simple genetic structure, and relatively high evolution rate [12,13,14,26]. Compared with single or multiple genes for phylogenetic analysis, mitogenomes provide relatively rich genetic information, which can better infer phylogenetic relationships and the degrees of relatedness among taxonomic groups [27,28].

Until now, studies of the genus *Atkinsoniella* have mainly focused on the description of new species, and few have been conducted on mitochondrial genomes and phylogenetic relationships. To better understand the relationships within the genus *Atkinsoniella*, 12 mitogenomes of *Atkinsoniella* were sequenced and annotated, and their characteristics were comparatively analyzed. In addition, the comprehensive phylogenetic relationship within the subfamily Cicadellinae was determined based on mitochondrial datasets.

## 2. Materials and Methods

### 2.1. Genome Organization

The adult specimens of 12 *Atkinsoniella* species (*A*. *aurantiaca* Cai *et* Kuoh, 1995; *A*. *curvata* Zhang *et* Kuoh 1993; *A*. *flavipenna* Li *et* Wang, 1992; *A*. *longiuscula* Feng *et* Zhang, 2015; *A*. *thalia* (Distant, 1918); *A*. *thaloidea* Young, 1986; *A*. *tiani* Yang, Meng *et* Li, 2017; *A*. *uniguttata* Li, 1993; *A*. *warpa* Yang, Meng *et* Li, 2017; *A*. *wui* Yang, Meng *et* Li, 2017; *A*. *xanthoabdomena* Yang, Meng *et* Li, 2017; *A*. *yunnanana* Yang, Meng *et* Li, 2017) used in this study were collected in China, and the detailed collection information is listed in Table S1. All of the fresh specimens were preserved in absolute ethanol immediately and stored at −20 °C in the laboratory before DNA extraction. Twelve *Atkinsoniella* species were identified through morphological characteristics as described by Yang et al. [3]. Total DNA was prepared from the head and thorax muscle tissues of a single adult individual using a DNeasy^®^ Tissue Kit (Qiagen, Hilden, Germany) according to the manufacturer’s protocol. The remainder of the specimens (wings and abdomens) were saved as vouchers and deposited at the Institute of Entomology, Guizhou University, Guiyang, China (GUGC).

### 2.2. PCR Amplification, Sequencing, and Assembly

The *COX1* fragments as the starting references for assembling mitochondrial genomes were amplified using the universal primer pairs LCO1490 (5′-GGTCAACAAATCATAAAGATATTGG-3′) and LCO2198 (5′-TAAACTTCAGGGTGACCAAAAAATCA -3′) [29]. Primer sequences were synthesized by Sangon Biotech Company (Shanghai, China). PCRs were performed in 25 µL reaction volumes in total, which contained 9 µL ddH_2_O, 13 µL 2X SanTaq PCR Master Mix (Sangon Biotech Company), 1 µL forward primer (0.01 mol/L), 1 µL reverse primer (0.01 mol/L), and 1 µL DNA template. The PCR amplification conditions were as follows: 94 °C for 5 min, followed by 35 cycles of 95 °C for 1 min, 51 °C for 1 min, elongation at 72 °C for 2 min, and a final extension step at 72 °C for 10 min. The PCR products were sequenced by Sangon Biotech Company. Total genomic DNA was used for library preparation and sequenced using the Illumina NovaSeq6000 platform with 150 bp paired-end reads at Berry Genomics (Beijing, China). Approximately 6 Gb clean data of each species were obtained and assembled using Getorganelle 1.7.4.1 [30] or NOVOPlasty 2.7.2 [31] with the corresponding *COX1* fragment of each species as a starting reference.

### 2.3. Mitogenome Annotation and Analysis

Initial annotations of these 12 mitogenomes were conducted with MitoZ 2.4-alpha [32] with the invertebrate mitochondrial genetic codes. The MITOS web server (http://mitos.bioinf.uni-leipzig.de/index.py, accessed on 1 July 2021) [33], tRNAscan-SE search server (http://lowelab.ucsc.edu/tRNAscan-SE/, accessed on 4 July 2021) [34] and open reading frames (ORFs) were employed to reconfirm the locations of tRNAs and PCGs with invertebrate mitochondrial genetic codes. Geneious Prime 2021.1.1 (https://www.geneious.com, accessed on 25 July 2021) was used to align the newly sequenced mitogenomes with the available mitogenomes of Cicadellidae in GenBank for further reconfirmation. The secondary structures of tRNA genes were predicted by the MITOS2 web server (http://mitos2.bioinf.uni-leipzig.de/index.py, accessed on 6 July 2021) and drawn with VARNAv3-92 [35], Rnaviz [36] and Adobe Illustrator SC6. Two rRNA genes were determined according to the locations of adjacent tRNA genes and compared with homologous mitogenome sequences of other Cicadellidae species using the MAFFT [37] algorithm in Geneious Prime 2021.1.1. Strand asymmetry was calculated using the following formulas: AT skew = [A − T]/[A + T] and GC skew = [G − C]/[G + C] [38]. The A + T content of nucleotide sequences, codon usage, and genetic distances (using the Kimura-2-parameter method) of PCGs were computed by MEGA 6.0 [39]. A heatmap of the relative synonymous codon usage (RSCU) values of PCGs (excluding stop codons) in the 12 newly sequenced mitogenomes was drawn using TBtools v1.09832 [40]. The rates of nonsynonymous substitutions (Ka) and synonymous substitutions (Ks) of 13 aligned PCGs were determined by DnaSP v6.12.03 [41]. The structure maps of mitogenomes were visualized using Geneious Prime 2021.1.1 and Adobe Illustrator SC6. These 12 newly sequenced mitogenomes were submitted to GenBank with accession numbers OL677863–OL677874.

### 2.4. Phylogenetic Analysis

In the phylogenetic analyses, the 12 newly obtained mitogenomes and 18 complete or nearly complete mitogenomes of Cicadellinae species available in GenBank (seven unannotated mitogenomes were annotated using the methods described in Section 2.3) were considered ingroup, and *Taharana fasciana* and *Iassus dorsalis* from Coelidiinae and Iassinae served as the outgroup (Table 1). Three datasets were concatenated for phylogenetic analysis: (1) cds_faa: amino acid sequences of the PCGs; (2) cds12_fna: first and second codon positions of the PCGs; (3) cds12_rrna: the first and the second codon positions of the PCGs and two rRNA genes. The tools used to obtain these datasets included SeqKit v.0.10.1 [42] (extract each gene from annotated mitogenomes), MAFFT v.7.3 [37] (align each gene), trimAl v1.4.1 [43] (remove gaps and ambiguous sites), and FASconCAT-G v.1.04 [44] (concatenate nucleotide and protein alignments). The partitioned datasets were analyzed using maximum-likelihood (ML) and Bayesian inferences (BI). ML analysis was reconstructed with IQ-TREE v.1.6.8 [45]. The partitioning schemes and the substitution models were estimated by ModelFinder [46] implemented in the IQ-TREE package with the Bayesian information criterion (BIC). Branch support was calculated with 10000 ultrafast bootstrap and 1000 SH-aLRT replicates. BI analysis was performed on MrBayes 3.2.6 [47] with the default settings by simulating four independent runs for 5–10 million generations with sampling every 1000 generations; after the average standard deviation of split frequencies fell below 0.01, the initial 25% of samples were discarded as burn-in. Partitioning schemes and the best-fitting substitution models were determined in PartitionFinder v.2.1.1 with BIC criterion and greedy-search algorithm [48].

## 3. Results

### 3.1. Genome Organization

**Figure 1:**
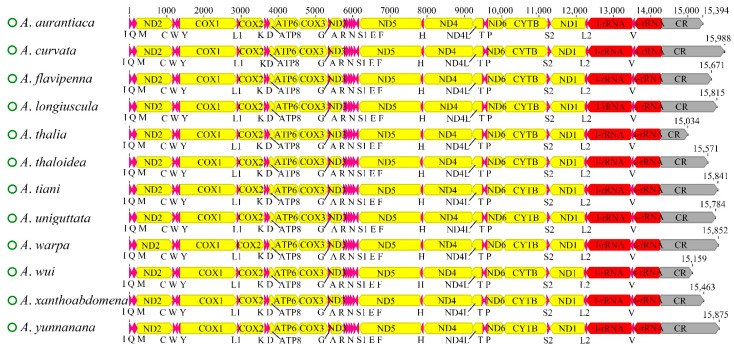
Characteristics of 12 newly sequenced mitochondrial genomes of *Atkinsoniella* species. Protein coding and ribosomal genes are shown with standard abbreviations. Transfer RNA (tRNA) genes are indicated using the IUPAC-IUB single letter amino acid codes (L1, CUN; L2, UUR; S2). The green circles in front of the species names represent circular mitochondrial genomes. The sharp angle of color blocks on the right and left indicates that the genes were on the J-strand and N-strand, respectively.

The complete mitochondrial genomes of the 12 newly sequenced *Atkinsoniella* species were obtained and deposited in the GenBank database (Table S1). All of the mitogenomes are closed circular molecules ranging from 15,034 bp (*A*. *thalia*) to 15,988 bp (*A*. *curvata*) in length, encoding 37 typical mitochondrial genes, including 13 protein-coding genes (PCGs), 22 transfer RNA genes (tRNAs), two ribosomal RNA genes (rRNAs), and a control region (CR). A total of 23 genes (9 PCGs and 14 tRNAs) were encoded on the majority strand (N-strand), while the remaining 14 genes (4 PCGs, 8 tRNAs, and 2 rRNAs) were encoded on the minority strand (J-strand). All 12 mitogenomes showed identical gene arrangements and orientations (Figure 1, Table S2), which were consistent with other Cicadellidae species. The mitogenomes had a significant bias toward A and T bases, with A + T contents ranging from 77.7 (*A*. *wui*) to 79.8 (*A*. *uniguttata*). All the AT skews were positive (ranging from 0.066 to 0.104), and all the GC skews were negative (ranging from −0.141 to −0.055) (Table S3), indicating that the occurrence of As and Cs was slightly higher than that of Ts and Gs. Variable intergenic spacers and overlap were commonly found in the 12 mitogenomes; the longest intergenic spacer was 4 bp between trnY and COX1, and the largest overlapping region was 8 bp, where trnC was included in trnW (Table S2).

### 3.2. Protein-Coding Genes and Codon Usage

**Figure 2:**
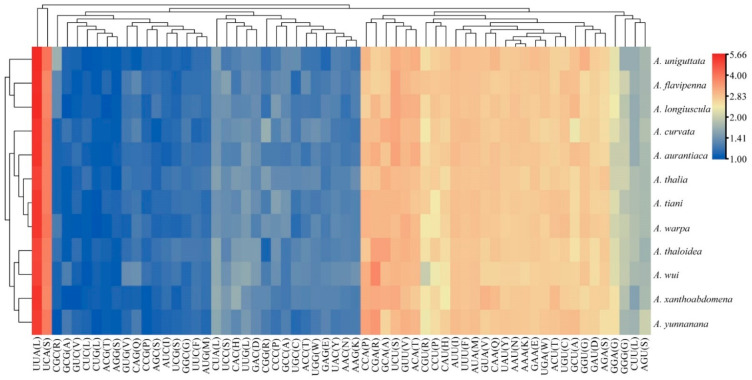
The relative synonymous codon usage (RSCU) of PCGs in the 12 newly sequenced mitogenomes. The x-axis and y-axis indicate the hierarchical clustering of codon frequencies and *Atkinsoniella* species, respectively.

Among the 13 PCGs of the 12 *Atkinsoniella* mitogenomes, nine were encoded on the J-strand, while the other four were encoded on the N-strand. Almost all of the newly sequenced mitogenomes exhibited similar start and stop codons. All the PCGs were initiated with typical ATN codons (ATT, ATG, ATC, or ATA), except for *ATP8* and *ND5* which were initiated with a TTG codon, and *ATP6* of three species (*A*. *aurantiaca*, *A*. *curvata*, and *A*. *flavipenna*) which started with GTG. Among the 13 PCGs of the 12 *Atkinsoniella* species, most PCGs terminated with complete termination codons TAA or TAG, whereas *COX2*, *COX3*, *ND5*, and *ND6* were terminated with a single T or TA residue (Table S4). Such incomplete terminal codons are widely recognized in invertebrate mitogenomes and are assumed to be generated by the polyadenylation process [14]. Meanwhile, the stop codon TAA occurred more frequently than TAG. Among the 13 PCGs, *ATP8* was the shortest (153 bp), and *ND5* (1675 bp) was the longest in all the 12 *Atkinsoniella* mitogenomes. The 13 PCGs were all 10,958 bp in length, encoding 3642 amino acids. The scope of A + T content in 13 PCGs ranged from 76.4 (*A*. *thalia*, *A*. *wui*) to 78.4 (*A*. *uniguttata*), and the A + T content of the third codon positions was much higher than that of the first and second codon positions (PCG1: 72.4–74.1, PCG2: 69.1–69.5, PCG3: 87.0–91.7) (Table S3), which is typical for the family Cicadellidae mitogenomes [59,60,61]. All 13 PCGs showed a negative AT skew (from −0.148 to −0.132) and a positive GC skew (from 0.010 to 0.044). The number of codon usage and relative synonymous codon usage (RSCU) of PCGs of the 12 determined *Atkinsoniella* mitogenomes were calculated and are shown in Figure 2 and Table S5, which show that the six most frequently used codons were AUU(I), UUA(L), UUU(F), AUA(M), UAU(Y), and AAU(N), while CUG(L), AGG(S), ACG(T), CUC(L), GUC(V), and GCG(A) were rarely used. Furthermore, the overall analysis of RSCU indicated that the codons ending up with T or A were more frequently used than those ending up with C or G (Figure 2).

**Figure 3:**
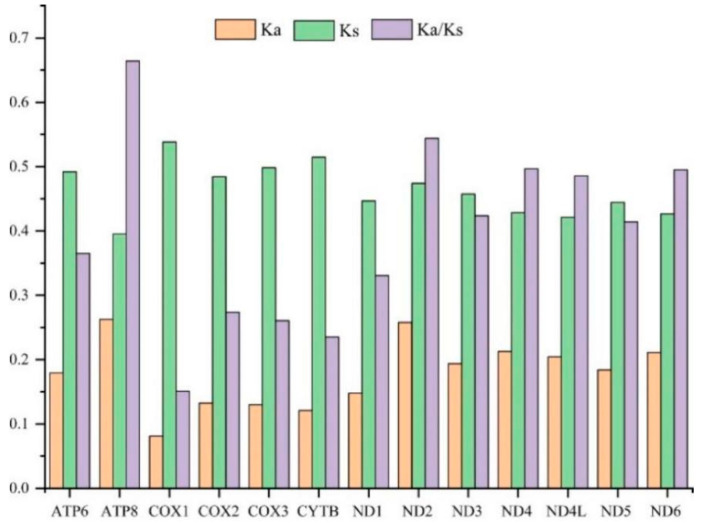
The evolutionary rate of each PCG in the mitogenomes of 32 Cicadellidae species in this study. Ka is the nonsynonymous nucleotide substitutions per nonsynonymous site, Ks is the synonymous nucleotide substitutions per synonymous site, and Ka/Ks is the ratio of the rate of nonsynonymous substitutions to the rate of synonymous substitutions.

**Figure 4:**
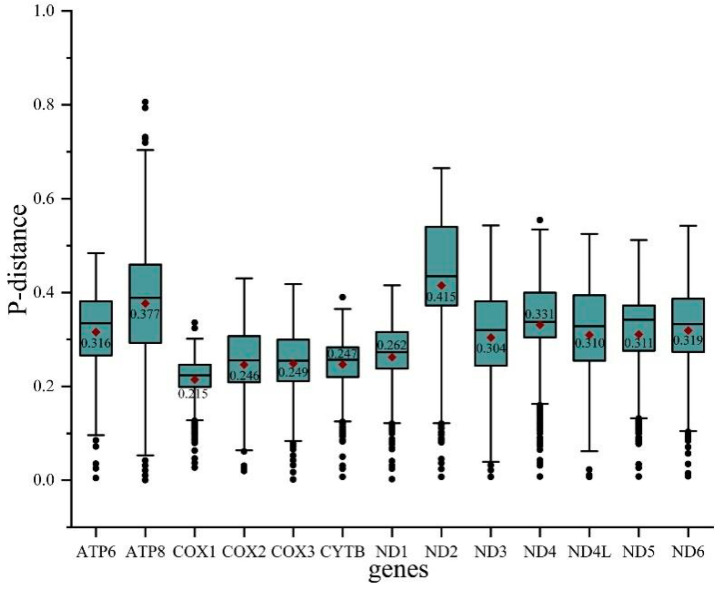
Boxplot showing the P-distance of each PCG in all 32 species studied. Outlier values are represented by black dots.

To evaluate the evolutionary patterns among 13 PCGs, the Ka (nonsynonymous substitutions), Ks (synonymous substitutions), and Ka/Ks (ω) values were estimated for each PCG of the 30 Cicadellinae species and two outgroups (Figure 3). The Ka and Ks values ranged from 0.08 (*COX1*) to 0.26 (*ATP8*) and 0.40 (*ATP8*) to 0.54 (*COX1*), respectively. The Ka/Ks values of all PCGs were less than one and increased from 0.15 for *COX1* to 0.66 for *ATP8*, indicating that the 13 PCGs were under purifying selection. Furthermore, *COX1*, which has been widely used as a marker for species identification, showed the lowest p-distance (0.215). The result was consistent with the studies of Ledrinae and Typhlocybinae (Cicadellidae) [62,63], as well as Seirinae (Collembola: Entomobryidae) [64], but different from that of Cleridae (Coleoptera: Cleroidea) [65] (Figure 4). While *ND2* possessed the greatest genetic distance with the value of 0.415 (Figure 4). The mitochondrial genes with the highest genetic divergence are variable among different insect groups [62,63,64,65] (Figure 4). In addition, the P-distances were measured based on the dataset of 13 PCGs across the 14 *Atkinsoniella* species to explore their sequence divergence (Table S6). According to previous studies, the intraspecific divergences are rarely greater than 2% and most are less than 1% [66],. This was also verified in studies of several insect groups, such as Lepidoptera [67], Piophilidae [68], Blattidae [69], Liposcelididae [70], etc. The recent study of leafhoppers based on *COX1* gene fragments also showed that the intraspecific divergence was less than 1% [71]. As shown in Table S6 of this study, the minimum intergeneric distance was 2.6% between *A*. *tiani* and *A*. *warpa*, followed by 3.5% between *A*. *flavipenna* and *A*. *longiuscula*., and the greatest variation was 21.4% between *A*. *wui* and *A*. *xanthonota*, indicating that the 12 yellow-winged *Atkinsoniella* species were separate species with close relationships.

### 3.3. Transfer and Ribosomal RNA Genes

**Figure 5:**
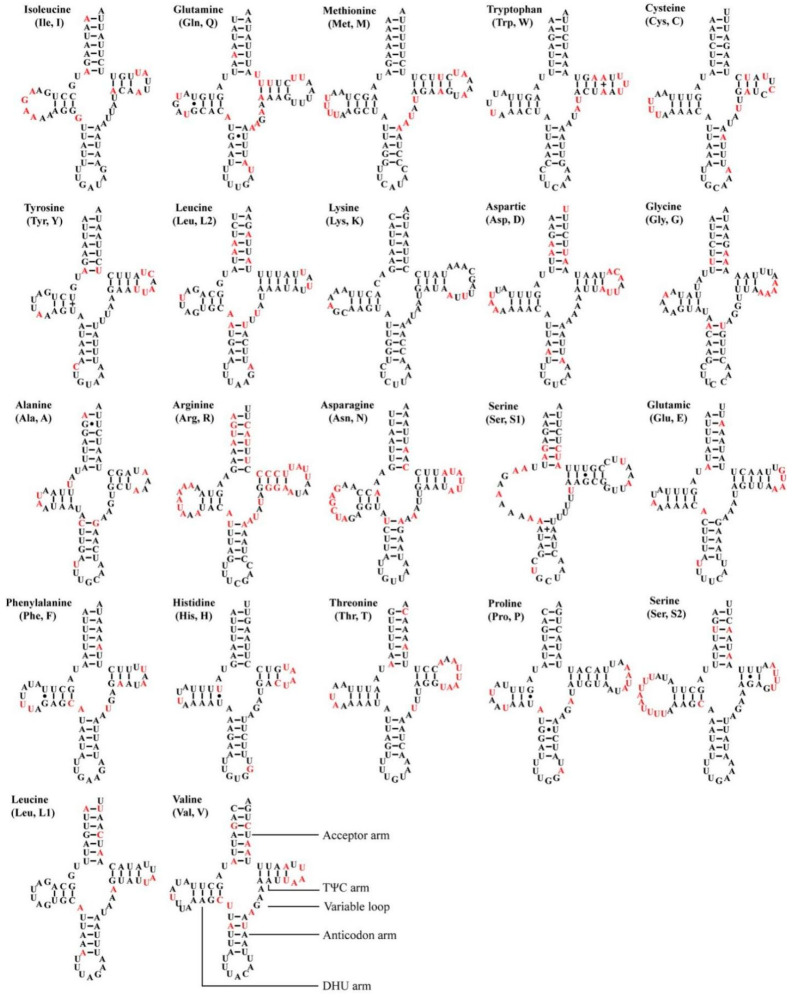
Predicted secondary cloverleaf structures for the tRNAs of the newly sequenced *Atkinsoniella* species. The tRNA arms are illustrated as for trnV. Dashes (–), solid dots (•), and pluses (+) indicate the Watson–Crick base pairings, G–U bonds, and mismatches, respectively. The conserved and variable sites among the 12 *Atkinsoniella* species are indicated using black and red, respectively.

All 12 *Atkinsoniella* mitogenomes contained the typical 22 transfer RNAs (tRNAs), 14 of which were encoded by the J-strand, and eight were encoded by the N-strand. The size of 22 tRNAs of the 12 mitogenomes ranged from 61 bp (*trnA* and *trnI* in *A*. *xanthoabdomena*, and *trnH* in all 12 *Atkinsoniella* mitogenomes) to 72 bp (*trnK* in all newly sequenced *Atkinsoniella* mitogenomes except for that of *A*. *uniguttata*) (Table S2). The total length of 22 tRNAs ranged from 1426 to 1443 bp, and the A + T content ranged from 79.9 to 81.4. The AT skew (0.003–0.021) and GC skew (0.110–0.166) of all tRNAs were both positive. All tRNAs showed typical cloverleaf structures, except for *trnS1*, which lacked a dihydrouridine (DHU) arm and was instead replaced by a simple loop (Figure 5), which is commonly found in Cicadellidae insect mitogenomes [49,58,61,72,73,74,75,76]. All the anticodons of each tRNA were identical to those reported for *Atkinsoniella* species [49]. In the predicted secondary structure, the nucleotides on the anticodon loop of all tRNAs were highly conserved for 7 bp compared with the size-variable DHU and TΨC loops.

Two rRNA genes (*s-rRNA* and *l-rRNA*) were recognized in all the sequenced mitogenomes, and both were encoded on the N-strand. The *l-rRNA* was located between *trnL1* and *trnV*, with lengths ranging from 1188 bp (*A*. *warpa*) to 1199 bp (*A*. *aurantiaca*, *A*. *longiuscula*). The *s-rRNA* was located between *trnV* and the control region, with lengths ranging from 730 bp (*A*. *flavipenna*, *A*. *longiuscula*, *A*. *uniguttata*, *A*. *xanthobdomena*) to 734 bp (*A*. *aurantiaca*). The A + T content of rRNAs ranged from 80.7% to 81.4%. Additionally, the two rRNAs showed a negative AT skew (−0.227–−0.142) and a positive GC skew (0.219–0.258).

### 3.4. Control Region

The control region, which is the longest noncoding region, contains controlling elements for replication and transcription. The control regions of the 12 *Atkinsoniella* mitogenomes were located between *s-rRNA* and *trnI* and were variable in length, ranging from 744 bp (*A*. *thalia*) to 1688 bp (*A*. *curvata*), with A + T contents ranging from 82.1% (*A*. *yunnanana*) to 88.6% (*A*. *tiani*). The AT skews of *A*. *aurantiaca*, *A*. *flavipenna*, and *A*. *longiuscula* were negative, with values of −0.02, −0.016, and −0.054, respectively. The remaining species shared a positive AT skew, ranging from 0.007 to 0.144. The GC skews were all negative, except for *A*. *aurantiaca*, which shared a positive value (0.046).

### 3.5. Phylogenetic Analyses

**Figure 6:**
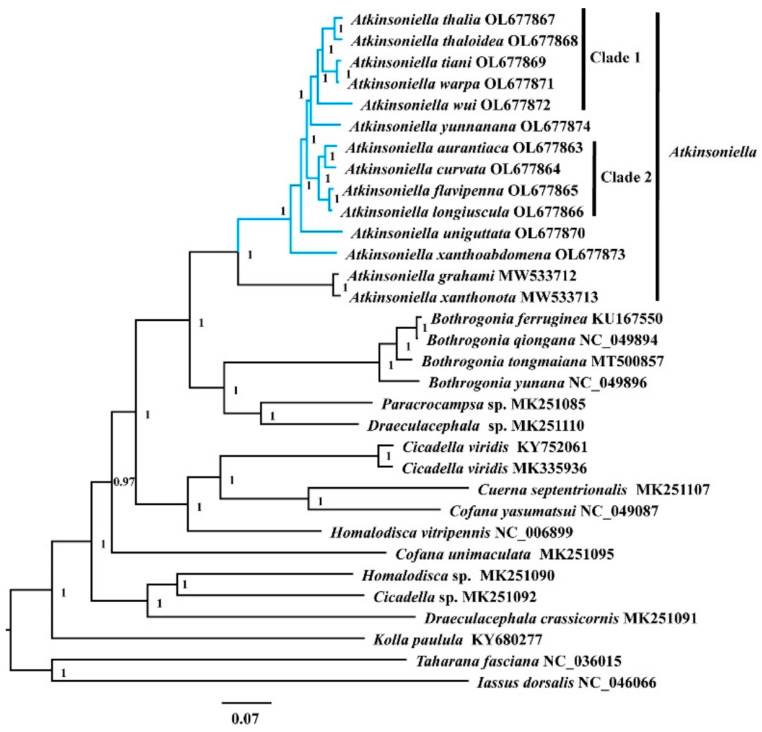
The phylogenetic relationships of Cicadellinae using the Bayesian inference (BI) method based on the concatenated amino-acid sequences of the PCG (cds_faa) dataset. Numbers on branches are posterior probabilities (PP). The blue clades represent the 12 newly sequenced *Atkinsoniella* mitogenomes in this study.

Phylogenetic relationships among 30 species of the subfamily Cicadellinae and two outgroup taxa from Coelidiinae and Iassinae were reconstructed based on 13 PCGs and two rRNAs using ML and BI analyses under the best partitioning scheme and models selected by Modelfinder (Table S7) and PartitionFinder (Table S8). Among the six resulting trees, the topology was highly identical within Cicadellinae and showed clearly separated branches of each species, with most nodes being highly supported in both ML and BI analyses, providing new insights into the phylogenetic relationship among *Atkinsoniella*, *Bothrogonia*, *Paracrocampsa*, *Draeculacephala*, *Cicadella*, *Cuerna*, *Cofana*, *Homalodisca*, and *Kolla* (Figure 6 and Figures S1–S5). All the phylogenetic analyses in this study strongly support that the genus *Atkinsoniella* was consistently recovered as a monophyletic group with strong support values and can be subdivided into two groups. The first group contained the 12 newly sequenced yellow-winged *Atkinsoniella* species. The second group was composed of sister taxa *A*. *grahami* and *A*. *xanthonota* [49]. The topological structure of the 12 newly sequenced species with highly similar morphological characteristics was completely congruent in all six phylogenetic trees, except for the differences observed in branches of *A*. *yunnanana*, *A*. *uniguttata*, and *A*. *xanthoabdomena*. The phylogenetic relationships within clade 1 and clade 2 were consistently recovered as (*A*. *wui* +((*A*. *tiani* + *A*. *warpa*) + (*A*. *thalia* + *A*. *thaloidea*))) and ((*A*. *aurantiaca* + *A*. *curvata*) + (*A*. *flavipenna* + *A*. *longiuscula*)) in all analyses, respectively (Figure 6 and Figures S1–S5). In the cds_faa_BI, cds12_fna_ML, and cds12_rrna_ML analyses, *A*. *yunnanana* and clade 1 clustered into one branch, forming a sister group with clade 2 (Figure 6 and Figures S1–S5). In the analyses of cds_faa_ML, cds12_fna_BI, and cds12_rrna_BI, *A*. *yunnanana* and clade 2 clustered into one branch, emerging as a sister group with clade 1. Additionally, *A*. *xanthoabdomena* was placed as the sister group to the remaining 11 yellow-winged species within all phylogenetic analyses, except that in the cds12_fna_BI analysis, the locations of *A*. *xanthoabdomena* and *A*. *uniguttata* were exchanged in cds12_fna_BI (Figure 6 and Figures S1–S5).

Traditionally, among the 12 yellow-winged *Atkinsoniella* species, some are difficult to identify due to their morphological similarities. For example, *A*. *thalia* and *A*. *thaloidea* are very similar in body color, markings, and even male genitalia; they can only be distinguished by the characteristics of their pygofer process. However, in this study, *A*. *thalia* and *A*. *thaloidea* were completely separated into two branches and strongly supported, demonstrating that the morphological characteristics that we currently use to identify *Atkinsoniella* species were proven to be reliable at the organelle-genome level. Furthermore, it also indicated that mitogenome sequences could be one of the effective methods to identify species with similar morphological characteristics and may contribute to the male and female correspondence of sexually dimorphic species as a means to shed light on the studies of insect biodiversity.

Meanwhile, the ML and BI analyses from this study also indicated that the genus *Bothrogonia* was coherently a monophyletic group, and the relationships within the genus were (((*B*. *ferruginea* + *B*. *qiongana*) +*B*. *tongmaiana*) + *B*. *yunana*), forming a sister group with the clade of *Paracrocampsa* sp. (MK251085) and *Draeculacephala* sp. (MK251110), which was completely concurrent with a previous study [52], except for the analysis based on the cds12_rrna dataset where *Draeculacephala* sp. (MK251110) was not included due to the lack of rRNA gene sequences. Meanwhile, *Cicadella viridis* emerged as a sister group with *Cuerna septentrionalis* and *Cofana yasumatsui* in all analyses. Unfortunately, the status of some species in the phylogenetic relationships remained questionable. For example, species from the genera *Cofana*, *Draeculacephala*, and *Homalodisca* were not clustered into a clade (e.g., *Cofana yasumatsui* and *C*. *unimaculata*, *Draeculacephala* sp. and *D*. *crassicornis*, *Homalodisca* sp. and *H*. *vitripennis*), showing that the three genera were polyphyletic groups. Furthermore, the Old World taxa (*Atkinsoniella*, *Bothrogonia*, *Cicadella*, *Cofana*, and *Kolla*) consistently formed well-supported clades with the New World taxa (Cuerna, *Draeculacephala*, Homalodisca, and *Paracrocampsa*) in all the phylogenetic trees. This was also observed in previous studies [51,52,54,55,77,78]. It is worth to notice that the genera included in the present study were all well-defined by previous studies. However, the species of the same genus from the New World in previous studies were not grouped together, strongly suggesting that some or all of these specimens may be misidentified. Thus, molecular data from additional individuals of each species is much needed in order to address these questions. Furthermore, additional specimens and molecular data are also needed to further clarify the phylogeny at the genus and species levels within the subfamily Cicadellinae.

### 3.6. Morphology

**Figure 7:**
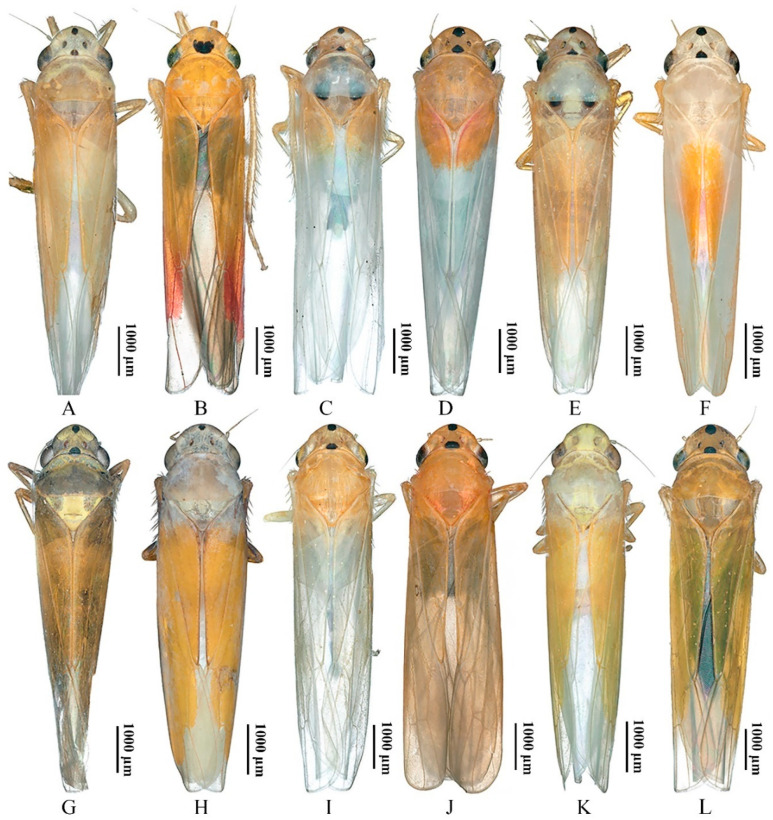
The dorsal views of 12 *Atkinsoniella* species. (**A**) *Atkinsoniella aurantiaca*; (**B**) *A*. *curvata*; (**C**) *A*. *flavipenna*; (**D**) *A*. *longiuscula*; (**E**) *A*. *thalia*; (**F**) *A*. *thaloidea*; (**G**) *A*. *tiani*; (**H**) *A*. *uniguttata*; (**I**) *A*. *warpa*; (**J**) *A*. *wui*; (**K**) *A*. *xanthoabdomena*; (**L**) *A*. *yunnanana*.

**Figure 8:**
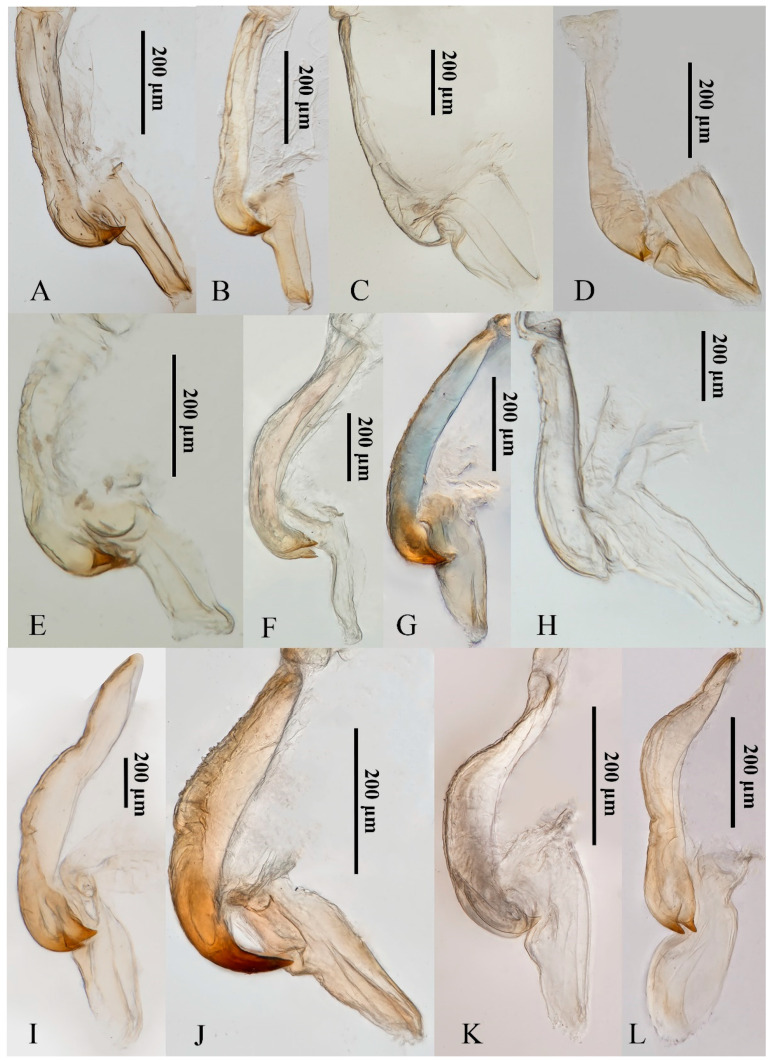
The paraphysis and aedeagus of 12 *Atkinsoniella* species. (**A**) *Atkinsoniella aurantiaca*; (**B**) *A*. *curvata*; (**C**) *A*. *flavipenna*; (**D**) *A*. *longiuscula*; (**E**) *A*. *thalia*; (**F**) *A*. *thaloidea*; (**G**) *A*. *tiani*; (**H**) *A*. *uniguttata*; (**I**) *A*. *warpa*; (**J**) *A*. *wui*; (**K**) *A*. *xanthoabdomena*; (**L**) *A*. *yunnanana*.

Although the 12 yellow-winged *Atkinsoniella* species are very similar in morphological characteristics, they can be distinguished by morphological characteristics. They share common morphological characteristics, with colour golden, pale yellow, pale white, or brown forewings without distinct spots or stripes, while there are some physical characteristics shown in Figure 7 that can be used to identify them in some degree. *A. aurantiaca*, *A*. *flavipenna*, *A*. *longiuscula*, *A*. *thalia*, *A*. *thaloidea*, *A*. *tiani*, *A*. *warpa*, *A*. *wui*, and *A*. *yunnanana* have a median black spot at the apical portion of the crown, and the median spot of basal portion is not V-shaped; *A*. *curvata* has no black spot at the apical portion of crown, and the spot of the basal portion is V-shaped; *A*. *uniguttata* has a median black spot at the apical portion of crown, but its basal portion does not have a spot; *A*. *xanthoabdomena* has a minimal black spot at the apical portion of crown in some individuals, and the basal portion without a spot; *A*. *flavipenna* and *A*. *thalia* have basal side spots on the scutellum. Moreover, their paraphysis and aedeagus in lateral view were shown in Figure 8 for further identification. The common characteristics of *Atkinsoniella* show apically forked, dorsally curved paraphysis, articulating with aedeagus. The major differences among the 12 species are reflected in the aedeagal size (*A*. *flavipenna* and *A*. *longiuscula* with aedeagus shorter and broader than others), and the different shape of the aedeagus and paraphysis. The other characteristics that can be used to identify them is provided in the following key:

Key to the 12 *Atkinsoniella* species1.Basal portion of crown without median black spots ........................................................... 2Basal portion of crown with a median black spot ................................................................ 32.Male pygofer process without branch ............................................................... *A. uniguttata*Male pygofer process with a small branch at subapex .......................... *A. xanthoabdomena*3.Apical portion of crown without black spots, the spot of basal portion V-shaped ................................................................................................................................................. *A. curvata*Apical portion of crown with a median black spot, the spot of basal portion not V-shaped ............................................................................................................................................... 44.Crown with large basal black spot, distinctly larger than the anterior median one ..................................................................................................................................................... *A. wui*Crown with small basal black spot, as big as or smaller than the anterior median one.................................................................................................................................................... 55.Scutellum with a black spot in each basal angle .................................................................. 6Scutellum without black spots ............................................................................................... 76Male pygofer process with apical 1/3 constricted and contorted; aedeagus broad andshort ........................................................................................................................ *A. flavipenna*Male pygofer process normal, with toothed process subapically; aedeagus stout and long,apex curved dorsad ..................................................................................................... *A. thalia*7.Male pygofer process with branch or toothed process subapically .................................. 8Male pygofer process without branch or process ................................................................ 98.Male pygofer process with membranous branch; paraphysis curved dorsad from medianportion, and with apical 1/3 curved posteroventrally ........................... *A. yunnanana*Male pygofer process with toothed process subapically, paraphysis curved not as above................................................................................................................................... *A. thaloidea*9.Male pygofer process foliated and contorted medially .................................................... 10Male pygofer process banding and not contorted medially ............................................ 1110.Forewing ivory, base orange-red; male pygofer rounded apically ............ *A. longiuscula*Forewing orange except apical membrane; male pygofer truncated apically ................................................................................................................................................... *A. aurantiaca*11.Male pygofer process not extending to apex of pygofer; aedeagus with transverse crackat ventral margin medially .......................................................................................... *A. tiani*Male pygofer process extending beyond apex of pygofer; aedeagus without transversecrack at ventral margin medially .............................................................................. *A. warpa*

## 4. Discussion

In this study, complete mitochondrial genomes of 12 yellow-winged *Atkinsoniella* species with similar morphological characteristics (*A*. *aurantiaca*, *A*. *curvata*, *A*. *flavipenna*, *A*. *longiuscula*, *A*. *thalia*, *A*. *thaloidea*, *A*. *tiani*, *A*. *uniguttata*, *A*. *warpa*, *A*. *wui*, *A*. *xanthoabdomena*, *A*. *yunnanana*) were newly sequenced and analyzed. The sizes of the 12 mitogenomes varied from 15,034 (*A*. *thalia*) to 15,988 bp (*A*. *curvata*), which was mainly due to the variation in the lengths of control regions. The comparative analysis showed that all 12 mitogenomes had similar structural characteristics and nucleotide compositions, and the gene order was consistent with hypothetical ancestral insects. All the *Atkinsoniella* mitogenomes contained 37 typical mitochondrial genes (13 PCGs, 22 tRNAs, two rRNAs) and a CR, and had a significant bias toward A and T bases. All PCGs were initiated with ATN codons, except for ATP8 and ND5 of the 12 *Atkinsoniella* species which were initiated with a TTG codon, and ATP6 of *A*. *aurantiaca*, *A*. *curvata*, and *A*. *flavipenna* which were initiated with GTG. Meanwhile, most PCGs were terminated with TAA or TAG, whereas COX2, COX3, ND5, and ND6 were terminated with a single T or TA residue.

Both ML and BI analyses based on the concatenated datasets of cds_faa, cds12_fna, and cds12_rrna indicated that the genus *Atkinsoniella* and *Bothrogonia* were consistently recovered as monophyletic groups. Additionally, *Atkinsoniella* can be subdivided into two groups. The first group contained the 12 newly sequenced yellow-winged *Atkinsoniella* species, which were clearly separated, with most nodes receiving high support values among the six phylogenetic trees. The second group was composed of *A*. *grahami* and *A*. *xanthonota*, whose forewings were black or dark brown with blood-red, orange-red or yellow-brown longitudinal stripes. The analyses also produced a well-resolved framework for the relationships within the subfamily Cicadellinae. The phylogenetic relationships with the genus *Atkinsoniella* indicated that mitogenomics is an effective method for identifying closely related species and may help to understand their phylogenetic and evolutionary relationships. However, some generic relationships within Cicadellinae remain uncertain, possibly due to the misidentification of the New World species and limited specimens of each genus. Further studies with more taxon samples from various animal geographic regions, richer molecular data, and a combined approach of mitochondrial and nuclear markers are needed to elucidate the phylogenetic and evolutionary relationships within Cicadellinae.

## Figures and Tables

**Table 1:** Mitochondrial genomes used for the phylogenetic analyses in this study.

Subfamily	Species	Size (bp)	A + T (%)	Accession Number	Reference
Cicadellinae	*Atkinsoniella aurantiaca*	15,394	79.2	OL677863	This Study
Cicadellinae	*Atkinsoniella curvata*	15,988	79.3	OL677864	This Study
Cicadellinae	*Atkinsoniella flavipenna*	15,671	79.1	OL677864	This Study
Cicadellinae	*Atkinsoniella grahami*	15,621	78.6	MW533712	[49]
Cicadellinae	*Atkinsoniella longiuscula*	15,815	79.2	OL677864	This Study
Cicadellinae	*Atkinsoniella thalia*	15,034	77.8	OL677864	This Study
Cicadellinae	*Atkinsoniella thaloidea*	15,571	78.2	OL677868	This Study
Cicadellinae	*Atkinsoniella tiani*	15,841	79.2	OL677868	This Study
Cicadellinae	*Atkinsoniella uniguttata*	15,784	79.8	OL677868	This Study
Cicadellinae	*Atkinsoniella warpa*	15,852	79.0	OL677868	This Study
Cicadellinae	*Atkinsoniella wui*	15,159	77.7	OL677868	This Study
Cicadellinae	*Atkinsoniella xanthoabdomena*	15,463	78.9	OL677868	This Study
Cicadellinae	*Atkinsoniella xanthonota*	15,895	78.4	MW533713	[49]
Cicadellinae	*Atkinsoniella yunnanana*	15,875	79.0	OL677874	This Study
Cicadellinae	*Bothrogonia ferruginea*	15,262	76.5	KU167550	[50]
Cicadellinae	*Bothrogonia qiongana*	15,788	76.9	NC_049894	[51]
Cicadellinae	*Bothrogonia tongmaiana*	15,539	76.5	MT500857	[52]
Cicadellinae	*Bothrogonia yunana*	15,585	76.4	NC_049896	[52]
Cicadellinae	*Cicadella* sp.	15,131	78.2	MK251092	[53]
Cicadellinae	*Cicadella viridis*	13,461	78.1	KY752061	Unpublished
Cicadellinae	*Cicadella viridis*	15,891	78.8	MK335936	[54]
Cicadellinae	*Cofana unimaculata*	14,865	77.6	MK251095	[53]
Cicadellinae	*Cofana yasumatsui*	15,019	77.2	NC_049087	[55]
Cicadellinae	*Cuerna septentrionalis*	12,420	77.1	MK251107	[53]
Cicadellinae	*Draeculacephala crassicornis*	15,214	76.9	MK251091	[53]
Cicadellinae	*Draeculacephala* sp.	12,400	75.5	MK251110	[53]
Cicadellinae	*Homalodisca* sp.	14,921	78.7	MK251090	[53]
Cicadellinae	*Homalodisca vitripennis*	15,304	78.4	NC_006899	Unpublished
Cicadellinae	*Kolla paulula*	13,579	73.3	KY680277	[56]
Cicadellinae	*Paracrocampsa* sp.	14,749	75.6	MK251085	[53]
Coelidiinae	*Taharana fasciana*	15,161	77.9	NC_036015	[57]
Iassinae	*Iassus dorsalis*	15,176	80.1	NC_046066	[58]

## Data Availability

Date available on request.

## Supplementary Materials

The following supporting information can be downloaded at: https://www.mdpi.com/article/10.3390/insects13030254/s1, Figure S1: Phylogenetic tree inferred by maximum likelihood (ML) based on the amino acids (cds_faa); Figure S2: Phylogenetic tree inferred by maximum likelihood (ML) based on the 1st and 2nd code position of protein-coding genes (cds12_fna); Figure S3: Phylogenetic tree inferred by Bayesian inference (BI) based on the 1st and 2nd code position of protein-coding genes (cds12_fna); Figure S4: Phylogenetic tree inferred by maximum likelihood (ML) based on the 1st and 2nd code position of protein coding genes concatenated rRNA (cds12_rrna); Figure S5: Phylogenetic tree inferred by Bayesian inference (BI) based on the 1st and 2nd code position of protein-coding genes concatenated rRNA (cds12_rrna), Table S1: Taxonomic information of newly sequenced species used for phylogenetic analysis in this study; Table S2: Annotations for the 12 newly sequenced *Atkinsoniella* mitogenomes; Table S3: Nucleotide composition of the 12 newly sequenced *Atkinsoniella* mitogenomes; Table S4: The start codons and stop codons of each protein-coding gene in the 12 *Atkinsoniella* mitogenomes; Table S5: The amount of codon usage of PCGs in the 12 newly sequenced *Atkinsoniella* mitogenomes; Table S6: Genetic distances of the 14 *Atkinsoniella* species in this study; Table S7: Best models were calculated by Modelfinder of cds_faa, cds12_fna, cds12_rrna datasets used in analysis; Table S8: Best models were calculated by PartitionFinder2 of cds_faa, cds12_fna, cds12_rrna datasets used in analysis.
