# Supplementary material for: Comparative Analysis of Mitochondrial Genomes among Twelve Sibling Species of the Genus Atkinsoniella Distant, 1908 (Hemiptera: Cicadellidae: Cicadellinae) and Phylogenetic Analysis
Source: Insects. 2022 Mar 3;13(3):254. doi: 10.3390/insects13030254 (PMC8953490; doi:10.3390/insects13030254)
Supplement: Supplementary file 1 [file insects-13-00254-s001.zip › new Table S5.pdf]

Table S5. The amount of codon usage of PCGs in the 12 newly sequenced *Atkinsoniella* mitogenomes

	A. aurantiaca	*A. curvata*	*A. flavipenna*	*A. longiuscula*	*A. thalia*	*A. thaloidea*	*A. tiani*	*A. uniguttata*	*A. warpa*	*A. wui*	*A. xanthoabdomena*	*A. yunnanana*
AAA(K)	97	103	101	99	93	98	97	107	98	93	104	104
AAC(N)	20	19	21	15	26	28	25	23	28	34	25	21
AAG(K)	13	10	15	15	21	16	16	11	16	24	14	14
AAU(N)	176	173	168	176	157	158	163	167	160	155	165	164
ACA(T)	82	75	77	86	71	73	67	78	73	75	67	77
ACC(T)	7	14	15	9	13	13	10	7	8	13	11	6
ACG(T)	1	2	5	0	3	3	4	2	1	2	2	2
ACU(T)	64	63	68	71	66	61	70	61	70	65	75	69
AGA(S)	74	70	71	69	82	81	76	75	78	76	76	69
AGC(S)	3	2	8	9	3	7	5	4	3	5	3	3
AGG(S)	3	2	1	2	1	2	4	2	4	1	4	2
AGU(S)	36	38	33	34	35	32	36	34	36	33	36	47
AUA(M)	310	321	315	312	301	299	322	323	320	322	304	322
AUC(I)	12	23	26	26	37	41	27	16	23	33	31	18
AUG(M)	28	23	27	26	38	33	23	25	25	28	27	18
AUU(I)	414	399	400	399	374	378	397	411	394	373	393	409
CAA(Q)	45	42	42	45	46	45	45	49	44	41	47	43
CAC(H)	9	13	7	10	16	16	11	9	13	19	22	11
CAG(Q)	4	7	8	5	4	5	5	1	6	10	2	6
CAU(H)	58	54	60	57	52	51	57	57	55	49	47	57
CCA(P)	57	55	51	48	52	51	59	57	59	61	62	66
CCC(P)	7	8	10	14	10	12	15	8	11	11	7	5
CCG(P)	4	3	4	2	5	3	2	3	5	5	1	1
CCU(P)	48	50	51	53	49	50	40	48	41	38	46	44
CGA(R)	26	23	20	24	22	28	24	21	24	33	26	30
CGC(R)	1	1	4	4	1	2	1	8	3	1	1	0
CGG(R)	3	8	3	2	6	1	3	2	5	3	4	3
CGU(R)	19	17	22	19	20	18	21	18	17	12	17	16
CUA(L)	37	36	36	34	42	48	33	40	34	48	52	47
CUC(L)	5	12	6	6	14	3	3	1	3	1	4	7

CUG(L)	3	4	2	6	4	8	1	2	0	6	5	1
CUU(L)	52	52	54	50	65	69	66	52	68	61	43	56
GAA(E)	71	66	70	67	71	69	70	74	70	65	68	67
GAC(D)	11	10	10	11	10	15	11	11	8	14	10	11
GAG(E)	9	13	9	12	8	10	11	4	9	13	12	12
GAU(D)	47	49	49	49	52	46	48	50	51	44	51	54
GCA(A)	40	48	37	38	48	54	45	40	46	46	49	37
GCC(A)	5	9	8	7	8	5	10	8	10	5	5	6
GCG(A)	2	4	2	0	1	1	1	2	1	5	0	5
GCU(A)	42	29	41	43	37	35	36	42	37	39	39	44
GGA(G)	46	55	59	62	48	53	53	56	49	54	68	68
GGC(G)	9	11	7	9	8	10	9	4	6	5	6	6
GGG(G)	46	45	48	41	48	43	40	29	45	44	39	33
GGU(G)	85	76	72	73	83	79	83	94	86	84	72	77
GUA(V)	72	74	68	67	79	70	71	72	74	70	86	72
GUC(V)	6	4	1	2	4	6	5	3	1	3	0	5
GUG(V)	6	9	9	7	9	6	7	4	3	16	3	5
GUU(V)	81	82	76	83	84	87	78	79	89	85	79	89
UAA(*)	8	8	7	7	8	8	8	9	8	8	8	7
UAC(Y)	24	20	28	27	27	27	30	26	32	35	28	20
UAG(*)	1	1	2	2	1	1	1	0	1	1	1	2
UAU(Y)	161	164	160	161	162	163	161	166	161	157	163	167
UCA(S)	132	133	124	126	133	133	135	140	141	126	125	135
UCC(S)	13	16	24	19	17	14	17	11	17	18	22	20
UCG(S)	5	6	3	4	10	12	8	5	4	9	3	5
UCU(S)	105	100	103	102	90	93	93	97	89	90	101	89
UGA(W)	78	73	82	82	75	78	81	83	76	78	78	81
UGC(C)	9	7	4	7	11	10	6	12	4	12	9	4
UGG(W)	13	18	9	9	16	13	10	8	15	13	14	10
UGU(C)	56	60	62	59	57	59	58	55	60	56	54	61
UUA(L)	363	361	370	378	328	321	348	374	348	326	355	336
UUC(F)	27	15	26	35	35	39	32	25	35	36	37	26
UUG(L)	40	35	31	26	36	40	40	28	37	48	33	37

UUU(F)	320	328	319	309	318	318	317	318	313	315	310	322
