# Supplementary material for: Comparative Analysis of Mitochondrial Genomes among Twelve Sibling Species of the Genus Atkinsoniella Distant, 1908 (Hemiptera: Cicadellidae: Cicadellinae) and Phylogenetic Analysis
Source: Insects. 2022 Mar 3;13(3):254. doi: 10.3390/insects13030254 (PMC8953490; doi:10.3390/insects13030254)
Supplement: Supplementary file 1 [file insects-13-00254-s001.zip › Table S1.pdf]

Table S1. The information of the 13 newly sequenced *Atkinsoniella* species in this study.

**Species**	**Accession number**	**Collected location**	**Collector**	**Date**	**Vocher number***	**Alttitute**
A.aurantiaca	OL677863	Baoshan, Yunnan, China,	Xian-Yi Wang	6-Aug-2018	GUGC-YN063-4	1692m
A. curvata	OL677864	Motuo, Xizang, China	Xiao-Fei Yu	29-Jul-2020	GUGC-XZ010-11	/
A. flavipenna	OL677865	Laohegou Nature Reserve, Sichuan, China	Mao-Fa Yang, Zai-Hua Yang, Xiao-Fei Yu, Yan Jiang	19-Aug-2018	GUGC-SC028-1	1890m
A. longiuscula	OL677866	Gaoligongshan, Yunnan, China	Jia-Jia Wang	7-Aug-2018	GUGC-YN003-5	/
A. thalia	OL677867	Longmen county, Sichuan, China	Mao-Fa Yang, Zai-Hua Yang, Xiao-Fei Yu, Yan Jiang	18-Aug-2018	GUGC-SC025-2	1794m
A. thaloidea	OL677868	Beibeng, Xizang, China	Xiao-Fei Yu	1-Aug-2020	GUGC-XZ013-1	/
A. tiani	OL677869	Baoshan, Yunnan, China,	Xian-Yi Wang	8-Aug-2018	GUGC-YN061-6	/
A. uniguttata	OL677870	Jianfengling, Hainan, China	Xiao-Li Xu	6-Jun-2019	GUGC-HN061-1	/
A. warpa	OL677871	Motuo, Xizang, China	Xiao-Fei Yu	29-Jul-2020	GUGC-XZ010-12	/
A. wui	OL677872	Motuo, Xizang, China	Jian-Yue Qiu	11-Aug-2017	GUGC-XZ002-1	/
A. xanthoabdomena	OL677873	Baoshan, Yunnan, China,	Lu Yang	5-Aug-2018	GUGC-YN023-4	/
A. yunnanana	OL677874	Gaoligongshan National Nature Reserve, Yunnan, China,	Lu Yang	7-Aug-2019	GUGC-YN029-1	2201m

Note: The slashes indicate that the altitudes were not recorded in the location information of the tested samples.

* Identification code permanently stored in Institute of Entomology, Guizhou University for recording and peer communication.
