# Supplementary material for: Comparative Analysis of Mitochondrial Genomes among Twelve Sibling Species of the Genus Atkinsoniella Distant, 1908 (Hemiptera: Cicadellidae: Cicadellinae) and Phylogenetic Analysis
Source: Insects. 2022 Mar 3;13(3):254. doi: 10.3390/insects13030254 (PMC8953490; doi:10.3390/insects13030254)
Supplement: Supplementary file 1 [file insects-13-00254-s001.zip › Table S2.pdf]

Table S2. Annotations for the 12 newly sequenced *Atkinsoniella* mitogenomes

Species	Gene	Minimum	Maximum	Length	Direction	Intergenic nucleotides	Start codon	Stop codon	Anticodon
A. aurantiaca	trnI	1	63	63	J	-3			GAU
A. aurantiaca	trnQ	61	128	68	N	-1			UUG
A. aurantiaca	trnM	128	195	68	J	0			CAU
A. aurantiaca	ND2	196	1167	972	J	-2	ATT	TAA	
A. aurantiaca	trnW	1166	1230	65	J	-8			UCA
A. aurantiaca	trnC	1223	1284	62	N	1			GCA
A. aurantiaca	trnY	1286	1349	64	N	4			GUA
A. aurantiaca	COX1	1354	2889	1536	J	1	ATG	TAA	
A. aurantiaca	trnL1	2891	2955	65	J	0			UAA
A. aurantiaca	COX2	2956	3634	679	J	0	ATT	T	
A. aurantiaca	trnK	3635	3706	72	J	-1			CUU
A. aurantiaca	trnD	3706	3769	64	J	0			GUC
A. aurantiaca	ATP8	3770	3922	153	J	-7	TTG	TAA	
A. aurantiaca	ATP6	3916	4569	654	J	0	GTG	TAA	
A. aurantiaca	COX3	4570	5348	779	J	-1	ATG	TA	
A. aurantiaca	trnG	5348	5411	64	J	0			UCC
A. aurantiaca	ND3	5412	5765	354	J	3	ATT	TAA	
A. aurantiaca	trnA	5769	5830	62	J	-2			UGC
A. aurantiaca	trnR	5829	5897	69	J	-2			UCG
A. aurantiaca	trnN	5896	5961	66	J	-1			GUU
A. aurantiaca	trnS1	5961	6024	64	J	1			GCU
A. aurantiaca	trnE	6026	6091	66	J	-1			UUC
A. aurantiaca	trnF	6091	6154	64	N	0			GAA

A. aurantiaca	ND5	6155	7829	1675	N	0	TTG	T	
A. aurantiaca	trnH	7830	7890	61	N	-1			GUG
A. aurantiaca	ND4	7890	9212	1323	N	-7	ATG	TAA	
A. aurantiaca	ND4L	9206	9487	282	N	2	ATG	TAA	
A. aurantiaca	trnT	9490	9554	65	J	0			UGU
A. aurantiaca	trnP	9555	9622	68	N	2			UGG
A. aurantiaca	ND6	9625	10,105	481	J	0	ATT	T	
A. aurantiaca	CYTB	10,106	11,242	1137	J	-2	ATG	TAG	
A. aurantiaca	trnS2	11,241	11,307	67	J	-1			UGA
A. aurantiaca	ND1	11,307	12,239	933	N	0	ATT	TAA	
A. aurantiaca	trnL2	12,240	12,302	63	N	0			UAG
A. aurantiaca	l-rRNA	12,303	13,501	1199	N	0			
A. aurantiaca	trnV	13,502	13,565	64	N	0			UAC
A. aurantiaca	s-rRNA	13,566	14,299	734	N	0			
A. aurantiaca	Control region	14,300	15,394	1095					
A. curvata	trnI	1	64	64	J	-3			GAU
A. curvata	trnQ	62	129	68	N	-1			UUG
A. curvata	trnM	129	196	68	J	0			CAU
A. curvata	ND2	197	1168	972	J	-2	ATT	TAA	
A. curvata	trnW	1167	1231	65	J	-8			UCA
A. curvata	trnC	1224	1285	62	N	1			GCA
A. curvata	trnY	1287	1350	64	N	4			GUA
A. curvata	COX1	1355	2890	1536	J	1	ATG	TAA	
A. curvata	trnL1	2892	2956	65	J	0			UAA
A. curvata	COX2	2957	3635	679	J	0	ATT	T	

A. curvata	trnK	3636	3707	72	J	-1			CUU
A. curvata	trnD	3707	3770	64	J	0			GUC
A. curvata	ATP8	3771	3923	153	J	-7	TTG	TAA	
A. curvata	ATP6	3917	4570	654	J	0	ATG	TAA	
A. curvata	COX3	4571	5349	779	J	-1	ATG	TA	
A. curvata	trnG	5349	5412	64	J	0			UCC
A. curvata	ND3	5413	5766	354	J	3	ATT	TAA	
A. curvata	trnA	5770	5831	62	J	-2			UGC
A. curvata	trnR	5830	5897	68	J	-2			UCG
A. curvata	trnN	5896	5961	66	J	-1			GUU
A. curvata	trnS1	5961	6026	66	J	1			GCU
A. curvata	trnE	6028	6093	66	J	-1			UUC
A. curvata	trnF	6093	6157	65	N	0			GAA
A. curvata	ND5	6158	7832	1675	N	0	TTG	T	
A. curvata	trnH	7833	7893	61	N	-1			GUG
A. curvata	ND4	7893	9215	1323	N	-7	ATG	TAA	
A. curvata	ND4L	9209	9490	282	N	2	ATG	TAA	
A. curvata	trnT	9493	9558	66	J	0			UGU
A. curvata	trnP	9559	9626	68	N	2			UGG
A. curvata	ND6	9629	10,109	481	J	0	ATT	T	
A. curvata	CYTB	10,110	11,246	1137	J	-2	ATG	TAG	
A. curvata	trnS2	11,245	11,312	68	J	-1			UGA
A. curvata	ND1	11,312	12,244	933	N	0	ATT	TAA	
A. curvata	trnL2	12,245	12,307	63	N	0			UAG
A. curvata	l-rRNA	12,308	13,503	1196	N	0			
A. curvata	trnV	13,504	13,567	64	N	0			UAC

A. curvata	s-rRNA	13,568	14,300	733	N	0			
A. curvata	Control region	14,301	15,988	1688					
A. flavipenna	trnI	1	65	65	J	-3			GAU
A. flavipenna	trnQ	63	130	68	N	-1			UUG
A. flavipenna	trnM	130	197	68	J	0			CAU
A. flavipenna	ND2	198	1169	972	J	-2	ATT	TAA	
A. flavipenna	trnW	1168	1232	65	J	-8			UCA
A. flavipenna	trnC	1225	1286	62	N	0			GCA
A. flavipenna	trnY	1287	1350	64	N	4			GUA
A. flavipenna	COX1	1355	2890	1536	J	1	ATG	TAA	
A. flavipenna	trnL1	2892	2956	65	J	0			UAA
A. flavipenna	COX2	2957	3635	679	J	0	ATT	T	
A. flavipenna	trnK	3636	3707	72	J	-1			CUU
A. flavipenna	trnD	3707	3770	64	J	0			GUC
A. flavipenna	ATP8	3771	3923	153	J	-7	TTG	TAG	
A. flavipenna	ATP6	3917	4570	654	J	0	GTG	TAA	
A. flavipenna	COX3	4571	5349	779	J	-1	ATG	TA	
A. flavipenna	trnG	5349	5412	64	J	0			UCC
A. flavipenna	ND3	5413	5766	354	J	3	ATC	TAA	
A. flavipenna	trnA	5770	5831	62	J	-1			UGC
A. flavipenna	trnR	5831	5898	68	J	-1			UCG
A. flavipenna	trnN	5898	5965	68	J	-1			GUU
A. flavipenna	trnS1	5965	6030	66	J	1			GCU
A. flavipenna	trnE	6032	6097	66	J	-1			UUC
A. flavipenna	trnF	6097	6160	64	N	0			GAA

A. flavipenna	ND5	6161	7835	1675	N	0	TTG	T	
A. flavipenna	trnH	7836	7896	61	N	-1			GUG
A. flavipenna	ND4	7896	9218	1323	N	-7	ATG	TAA	
A. flavipenna	ND4L	9212	9493	282	N	2	ATG	TAA	
A. flavipenna	trnT	9496	9560	65	J	0			UGU
A. flavipenna	trnP	9561	9628	68	N	2			UGG
A. flavipenna	ND6	9631	10,111	481	J	0	ATT	T	
A. flavipenna	CYTB	10,112	11,248	1137	J	-2	ATG	TAG	
A. flavipenna	trnS2	11,247	11,316	70	J	-1			UGA
A. flavipenna	ND1	11,316	12,248	933	N	0	ATT	TAA	
A. flavipenna	trnL2	12,249	12,311	63	N	0			UAG
A. flavipenna	l-rRNA	12,312	13,508	1197	N	0			
A. flavipenna	trnV	13,509	13,573	65	N	0			UAC
A. flavipenna	s-rRNA	13,574	14,303	730	N	0			
A. flavipenna	Control region	14,304	15,671	1368					
A. longiuscula	trnI	1	65	65	J	-3			GAU
A. longiuscula	trnQ	63	130	68	N	-1			UUG
A. longiuscula	trnM	130	197	68	J	0			CAU
A. longiuscula	ND2	198	1169	972	J	-2	ATT	TAA	
A. longiuscula	trnW	1168	1232	65	J	-8			UCA
A. longiuscula	trnC	1225	1286	62	N	0			GCA
A. longiuscula	trnY	1287	1350	64	N	4			GUA
A. longiuscula	COX1	1355	2890	1536	J	1	ATG	TAA	
A. longiuscula	trnL1	2892	2956	65	J	0			UAA
A. longiuscula	COX2	2957	3635	679	J	0	ATT	T	

A. longiuscula	trnK	3636	3707	72	J	-1			CUU
A. longiuscula	trnD	3707	3770	64	J	0			GUC
A. longiuscula	ATP8	3771	3923	153	J	-7	TTG	TAG	
A. longiuscula	ATP6	3917	4570	654	J	0	GTG	TAA	
A. longiuscula	COX3	4571	5349	779	J	-1	ATG	TA	
A. longiuscula	trnG	5349	5412	64	J	0			UCC
A. longiuscula	ND3	5413	5766	354	J	3	ATC	TAA	
A. longiuscula	trnA	5770	5831	62	J	-1			UGC
A. longiuscula	trnR	5831	5898	68	J	-1			UCG
A. longiuscula	trnN	5898	5965	68	J	-1			GUU
A. longiuscula	trnS1	5965	6030	66	J	1			GCU
A. longiuscula	trnE	6032	6097	66	J	-1			UUC
A. longiuscula	trnF	6097	6160	64	N	0			GAA
A. longiuscula	ND5	6161	7835	1675	N	0	TTG	T	
A. longiuscula	trnH	7836	7896	61	N	-1			GUG
A. longiuscula	ND4	7896	9218	1323	N	-7	ATG	TAA	
A. longiuscula	ND4L	9212	9493	282	N	2	ATG	TAA	
A. longiuscula	trnT	9496	9560	65	J	0			UGU
A. longiuscula	trnP	9561	9628	68	N	2			UGG
A. longiuscula	ND6	9631	10111	481	J	0	ATT	T	
A. longiuscula	CYTB	10,112	11,248	1137	J	-2	ATG	TAG	
A. longiuscula	trnS2	11,247	11,316	70	J	-1			UGA
A. longiuscula	ND1	11,316	12,248	933	N	0	ATT	TAA	
A. longiuscula	trnL2	12,249	12,311	63	N	0			UAG
A. longiuscula	l-rRNA	12,312	13,510	1199	N	0			
A. longiuscula	trnV	13,511	13,575	65	N	0			UAC

A. longiuscula	s-rRNA	13,576	14,305	730	N	0			
A. longiuscula	Control region	14,306	15,815	1510					
A. thalia	trnI	1	64	64	J	-3			GAU
A. thalia	trnQ	62	129	68	N	-1			UUG
A. thalia	trnM	129	197	69	J	0			CAU
A. thalia	ND2	198	1169	972	J	-2	ATT	TAA	
A. thalia	trnW	1168	1232	65	J	-8			UCA
A. thalia	trnC	1225	1287	63	N	1			GCA
A. thalia	trnY	1289	1352	64	N	3			GUA
A. thalia	COX1	1356	2891	1536	J	1	ATG	TAA	
A. thalia	trnL1	2893	2957	65	J	0			UAA
A. thalia	COX2	2958	3636	679	J	0	ATT	T	
A. thalia	trnK	3637	3708	72	J	-1			CUU
A. thalia	trnD	3708	3771	64	J	-1			GUC
A. thalia	ATP8	3771	3923	153	J	-7	TTG	TAA	
A. thalia	ATP6	3917	4570	654	J	0	ATG	TAA	
A. thalia	COX3	4571	5349	779	J	-1	ATG	TA	
A. thalia	trnG	5349	5412	64	J	0			UCC
A. thalia	ND3	5413	5766	354	J	3	ATC	TAA	
A. thalia	trnA	5770	5831	62	J	-1			UGC
A. thalia	trnR	5831	5895	65	J	-1			UCG
A. thalia	trnN	5895	5961	67	J	-1			GUU
A. thalia	trnS1	5961	6026	66	J	1			GCU
A. thalia	trnE	6028	6093	66	J	-1			UUC
A. thalia	trnF	6093	6155	63	N	0			GAA

A. thalia	ND5	6156	7830	1675	N	0	TTG	T	
A. thalia	trnH	7831	7891	61	N	-1			GUG
A. thalia	ND4	7891	9213	1323	N	-7	ATG	TAA	
A. thalia	ND4L	9207	9488	282	N	2	ATG	TAA	
A. thalia	trnT	9491	9556	66	J	0			UGU
A. thalia	trnP	9557	9624	68	N	2			UGG
A. thalia	ND6	9627	10,107	481	J	0	ATT	T	
A. thalia	CYTB	10,108	11,244	1137	J	-2	ATG	TAG	
A. thalia	trnS2	11,243	11,309	67	J	-1			UGA
A. thalia	ND1	11,309	12,241	933	N	0	ATT	TAA	
A. thalia	trnL2	12,242	12,304	63	N	0			UAG
A. thalia	l-rRNA	12,305	13,494	1190	N	0			
A. thalia	trnV	13,495	13,558	64	N	0			UAC
A. thalia	s-rRNA	13,559	14,290	732	N	0			
A. thalia	Control region	14,291	15,034	744					
A. thaloidea	trnI	1	63	63	J	-3			GAU
A. thaloidea	trnQ	61	128	68	N	-1			UUG
A. thaloidea	trnM	128	196	69	J	0			CAU
A. thaloidea	ND2	197	1168	972	J	-2	ATT	TAA	
A. thaloidea	trnW	1167	1231	65	J	-8			UCA
A. thaloidea	trnC	1224	1286	63	N	1			GCA
A. thaloidea	trnY	1288	1351	64	N	3			GUA
A. thaloidea	COX1	1355	2890	1536	J	0	ATG	TAA	
A. thaloidea	trnL1	2891	2955	65	J	0			UAA
A. thaloidea	COX2	2956	3634	679	J	0	ATT	T	

A. thaloidea	trnK	3635	3706	72	J	-1				CUU
A. thaloidea	trnD	3706	3769	64	J	-1				GUC
A. thaloidea	ATP8	3769	3921	153	J	-7		TTG	TAA	
A. thaloidea	ATP6	3915	4568	654	J	0		ATG	TAA	
A. thaloidea	COX3	4569	5347	779	J	-1		ATG	TA	
A. thaloidea	trnG	5347	5410	64	J	0				UCC
A. thaloidea	ND3	5411	5764	354	J	3		ATC	TAA	
A. thaloidea	trnA	5768	5829	62	J	-1				UGC
A. thaloidea	trnR	5829	5893	65	J	-1				UCG
A. thaloidea	trnN	5893	5959	67	J	-1				GUU
A. thaloidea	trnS1	5959	6024	66	J	1				GCU
A. thaloidea	trnE	6026	6091	66	J	-1				UUC
A. thaloidea	trnF	6091	6153	63	N	0				GAA
A. thaloidea	ND5	6154	7828	1675	N	0		TTG	T	
A. thaloidea	trnH	7829	7889	61	N	-1				GUG
A. thaloidea	ND4	7889	9211	1323	N	-7		ATG	TAA	
A. thaloidea	ND4L	9205	9486	282	N	2		ATG	TAA	
A. thaloidea	trnT	9489	9554	66	J	0				UGU
A. thaloidea	trnP	9555	9622	68	N	2				UGG
A. thaloidea	ND6	9625	10,105	481	J	0		ATT	T	
A. thaloidea	CYTB	10,106	11,242	1137	J	-2		ATG	TAG	
A. thaloidea	trnS2	11,241	11,307	67	J	-1				UGA
A. thaloidea	ND1	11,307	12,239	933	N	0		ATT	TAA	
A. thaloidea	trnL2	12,240	12,302	63	N	0				UAG
A. thaloidea	l-rRNA	12,303	13,494	1192	N	0				
A. thaloidea	trnV	13,495	13,558	64	N	0				UAC

A. thaloidea	s-rRNA	13,559	14,289	731	N	0			
A. thaloidea	Control region	14,290	15,571	1282					
A. tiani	trnI	1	63	63	J	-3			GAU
A. tiani	trnQ	61	128	68	N	-1			UUG
A. tiani	trnM	128	196	69	J	0			CAU
A. tiani	ND2	197	1168	972	J	-2	ATT	TAA	
A. tiani	trnW	1167	1231	65	J	-8			UCA
A. tiani	trnC	1224	1285	62	N	1			GCA
A. tiani	trnY	1287	1350	64	N	3			GUA
A. tiani	COX1	1354	2889	1536	J	1	ATG	TAA	
A. tiani	trnL1	2891	2955	65	J	0			UAA
A. tiani	COX2	2956	3634	679	J	0	ATT	T	
A. tiani	trnK	3635	3706	72	J	-1			CUU
A. tiani	trnD	3706	3769	64	J	-1			GUC
A. tiani	ATP8	3769	3921	153	J	-7	TTG	TAA	
A. tiani	ATP6	3915	4568	654	J	0	ATG	TAA	
A. tiani	COX3	4569	5347	779	J	-1	ATG	TA	
A. tiani	trnG	5347	5410	64	J	0			UCC
A. tiani	ND3	5411	5764	354	J	3	ATT	TAA	
A. tiani	trnA	5768	5829	62	J	-1			UGC
A. tiani	trnR	5829	5893	65	J	-1			UCG
A. tiani	trnN	5893	5960	68	J	-1			GUU
A. tiani	trnS1	5960	6025	66	J	1			GCU
A. tiani	trnE	6027	6092	66	J	-1			UUC
A. tiani	trnF	6092	6154	63	N	0			GAA

A. tiani	ND5	6155	7829	1675	N	0	TTG	T	
A. tiani	trnH	7830	7890	61	N	-1			GUG
A. tiani	ND4	7890	9212	1323	N	-7	ATG	TAA	
A. tiani	ND4L	9206	9487	282	N	2	ATG	TAA	
A. tiani	trnT	9490	9554	65	J	0			UGU
A. tiani	trnP	9555	9622	68	N	2			UGG
A. tiani	ND6	9625	10,105	481	J	0	ATT	T	
A. tiani	CYTB	10,106	11,242	1137	J	-2	ATG	TAG	
A. tiani	trnS2	11,241	11,309	69	J	-1			UGA
A. tiani	ND1	11,309	12,241	933	N	0	ATT	TAA	
A. tiani	trnL2	12,242	12,304	63	N	0			UAG
A. tiani	l-rRNA	12,305	13,493	1189	N	0			
A. tiani	trnV	13,494	13,557	64	N	0			UAC
A. tiani	s-rRNA	13,558	14,288	731	N	0			
A. tiani	Control region	14,289	15,841	1553					
A. uniguttata	trnI	1	63	63	J	-3			GAU
A. uniguttata	trnQ	61	129	69	N	1			UUG
A. uniguttata	trnM	131	200	70	J	0			CAU
A. uniguttata	ND2	201	1172	972	J	-2	ATA	TAA	
A. uniguttata	trnW	1171	1235	65	J	-8			UCA
A. uniguttata	trnC	1228	1289	62	N	1			GCA
A. uniguttata	trnY	1291	1354	64	N	4			GUA
A. uniguttata	COX1	1359	2894	1536	J	0	ATG	TAA	
A. uniguttata	trnL1	2895	2959	65	J	0			UAA
A. uniguttata	COX2	2960	3638	679	J	0	ATT	T	

A. uniguttata	trnK	3639	3709	71	J	-1			CUU
A. uniguttata	trnD	3709	3773	65	J	-1			GUC
A. uniguttata	ATP8	3773	3925	153	J	-7	TTG	TAA	
A. uniguttata	ATP6	3919	4572	654	J	0	ATG	TAA	
A. uniguttata	COX3	4573	5351	779	J	-1	ATG	TA	
A. uniguttata	trnG	5351	5415	65	J	0			UCC
A. uniguttata	ND3	5416	5769	354	J	3	ATA	TAA	
A. uniguttata	trnA	5773	5834	62	J	-1			UGC
A. uniguttata	trnR	5834	5900	67	J	-2			UCG
A. uniguttata	trnN	5899	5965	67	J	-1			GUU
A. uniguttata	trnS1	5965	6030	66	J	-1			GCU
A. uniguttata	trnE	6030	6094	65	J	-1			UUC
A. uniguttata	trnF	6094	6157	64	N	0			GAA
A. uniguttata	ND5	6158	7832	1675	N	0	TTG	T	
A. uniguttata	trnH	7833	7893	61	N	-1			GUG
A. uniguttata	ND4	7893	9215	1323	N	-7	ATG	TAA	
A. uniguttata	ND4L	9209	9490	282	N	2	ATG	TAA	
A. uniguttata	trnT	9493	9557	65	J	0			UGU
A. uniguttata	trnP	9558	9624	67	N	2			UGG
A. uniguttata	ND6	9627	10,107	481	J	0	ATT	T	
A. uniguttata	CYTB	10,108	11,244	1137	J	6	ATG	TAG	
A. uniguttata	trnS2	11,251	11,312	62	J	-1			UGA
A. uniguttata	ND1	11,312	12,244	933	N	0	ATT	TAA	
A. uniguttata	trnL2	12,245	12,307	63	N	0			UAG
A. uniguttata	l-rRNA	12,308	13,496	1189	N	0			
A. uniguttata	trnV	13,497	13,560	64	N	0			UAC

A. uniguttata	s-rRNA	13,561	14,290	730	N	0			
A. uniguttata	Control region	14,291	15,784	1494					
A. warpa	trnI	1	63	63	J	-3			GAU
A. warpa	trnQ	61	128	68	N	-1			UUG
A. warpa	trnM	128	196	69	J	0			CAU
A. warpa	ND2	197	1168	972	J	-2	ATT	TAA	
A. warpa	trnW	1167	1231	65	J	-8			UCA
A. warpa	trnC	1224	1285	62	N	1			GCA
A. warpa	trnY	1287	1350	64	N	3			GUA
A. warpa	COX1	1354	2889	1536	J	1	ATG	TAA	
A. warpa	trnL1	2891	2955	65	J	0			UAA
A. warpa	COX2	2956	3634	679	J	0	ATT	T	
A. warpa	trnK	3635	3706	72	J	-1			CUU
A. warpa	trnD	3706	3769	64	J	-1			GUC
A. warpa	ATP8	3769	3921	153	J	-7	TTG	TAA	
A. warpa	ATP6	3915	4568	654	J	0	ATG	TAA	
A. warpa	COX3	4569	5347	779	J	-1	ATG	TA	
A. warpa	trnG	5347	5410	64	J	0			UCC
A. warpa	ND3	5411	5764	354	J	3	ATT	TAA	
A. warpa	trnA	5768	5829	62	J	-1			UGC
A. warpa	trnR	5829	5893	65	J	-1			UCG
A. warpa	trnN	5893	5960	68	J	-1			GUU
A. warpa	trnS1	5960	6025	66	J	1			GCU
A. warpa	trnE	6027	6092	66	J	-1			UUC
A. warpa	trnF	6092	6154	63	N	0			GAA

A. warpa	ND5	6155	7829	1675	N	0	TTG	T	
A. warpa	trnH	7830	7890	61	N	-1			GUG
A. warpa	ND4	7890	9212	1323	N	-7	ATG	TAA	
A. warpa	ND4L	9206	9487	282	N	2	ATG	TAA	
A. warpa	trnT	9490	9554	65	J	0			UGU
A. warpa	trnP	9555	9622	68	N	2			UGG
A. warpa	ND6	9625	10,105	481	J	0	ATT	T	
A. warpa	CYTB	10,106	11,242	1137	J	-2	ATG	TAG	
A. warpa	trnS2	11,241	11,309	69	J	-1			UGA
A. warpa	ND1	11,309	12,241	933	N	0	ATT	TAA	
A. warpa	trnL2	12,242	12,304	63	N	0			UAG
A. warpa	l-rRNA	12,305	13,492	1188	N	0			
A. warpa	trnV	13,493	13,556	64	N	0			UAC
A. warpa	s-rRNA	13,557	14,287	731	N	0			
A. warpa	Control region	14,288	15,852	1565					
A. wui	trnI	1	62	62	J	-3			GAU
A. wui	trnQ	60	127	68	N	-1			UUG
A. wui	trnM	127	195	69	J	0			CAU
A. wui	ND2	196	1167	972	J	-2	ATT	TAA	
A. wui	trnW	1166	1229	64	J	-8			UCA
A. wui	trnC	1222	1284	63	N	0			GCA
A. wui	trnY	1285	1347	63	N	3			GUA
A. wui	COX1	1351	2886	1536	J	0	ATG	TAA	
A. wui	trnL1	2887	2951	65	J	0			UAA
A. wui	COX2	2952	3630	679	J	0	ATT	T	

A. wui	trnK	3631	3702	72	J	-1			CUU
A. wui	trnD	3702	3764	63	J	-1			GUC
A. wui	ATP8	3764	3916	153	J	-7	TTG	TAA	
A. wui	ATP6	3910	4563	654	J	0	ATG	TAA	
A. wui	COX3	4564	5342	779	J	-1	ATG	TA	
A. wui	trnG	5342	5405	64	J	0			UCC
A. wui	ND3	5406	5759	354	J	3	ATC	TAA	
A. wui	trnA	5763	5824	62	J	-2			UGC
A. wui	trnR	5823	5887	65	J	-2			UCG
A. wui	trnN	5886	5951	66	J	-1			GUU
A. wui	trnS1	5951	6016	66	J	1			GCU
A. wui	trnE	6018	6083	66	J	-1			UUC
A. wui	trnF	6083	6145	63	N	0			GAA
A. wui	ND5	6146	7820	1675	N	0	TTG	T	
A. wui	trnH	7821	7881	61	N	-1			GUG
A. wui	ND4	7881	9203	1323	N	-7	ATG	TAA	
A. wui	ND4L	9197	9478	282	N	2	ATG	TAA	
A. wui	trnT	9481	9545	65	J	0			UGU
A. wui	trnP	9546	9614	69	N	2			UGG
A. wui	ND6	9617	10,097	481	J	0	ATT	T	
A. wui	CYTB	10,098	11,234	1137	J	-2	ATG	TAG	
A. wui	trnS2	11,233	11,295	63	J	-1			UGA
A. wui	ND1	11,295	12,227	933	N	0	ATT	TAA	
A. wui	trnL2	12,228	12,290	63	N	0			UAG
A. wui	l-rRNA	12,291	13,480	1190	N	0			
A. wui	trnV	13,481	13,544	64	N	0			UAC

A. wui	s-rRNA	13,545	14,276	732	N	0			
A. wui	Control region	14,277	15,159	883					
A. xanthoabdomena	trnI	1	61	61	J	-3			GAU
A. xanthoabdomena	trnQ	59	126	68	N	-1			UUG
A. xanthoabdomena	trnM	126	194	69	J	0			CAU
A. xanthoabdomena	ND2	195	1166	972	J	-2	ATT	TAA	
A. xanthoabdomena	trnW	1165	1229	65	J	-8			UCA
A. xanthoabdomena	trnC	1222	1283	62	N	1			GCA
A. xanthoabdomena	trnY	1285	1348	64	N	4			GUA
A. xanthoabdomena	COX1	1353	2888	1536	J	1	ATG	TAA	
A. xanthoabdomena	trnL1	2890	2954	65	J	0			UAA
A. xanthoabdomena	COX2	2955	3633	679	J	0	ATT	T	
A. xanthoabdomena	trnK	3634	3705	72	J	-1			CUU
A. xanthoabdomena	trnD	3705	3769	65	J	-1			GUC
A. xanthoabdomena	ATP8	3769	3921	153	J	-7	TTG	TAA	
A. xanthoabdomena	ATP6	3915	4568	654	J	0	ATG	TAA	
A. xanthoabdomena	COX3	4569	5347	779	J	-1	ATG	TA	
A. xanthoabdomena	trnG	5347	5410	64	J	0			UCC
A. xanthoabdomena	ND3	5411	5764	354	J	3	ATT	TAA	
A. xanthoabdomena	trnA	5768	5828	61	J	-1			UGC
A. xanthoabdomena	trnR	5828	5893	66	J	-6			UCG
A. xanthoabdomena	trnN	5888	5953	66	J	-1			GUU
A. xanthoabdomena	trnS1	5953	6018	66	J	1			GCU
A. xanthoabdomena	trnE	6020	6084	65	J	-1			UUC
A. xanthoabdomena	trnF	6084	6146	63	N	0			GAA

A. xanthoabdomena	ND5	6147	7821	1675	N	0	TTG	T	
A. xanthoabdomena	trnH	7822	7882	61	N	-1			GUG
A. xanthoabdomena	ND4	7882	9204	1323	N	-7	ATG	TAA	
A. xanthoabdomena	ND4L	9198	9479	282	N	2	ATG	TAA	
A. xanthoabdomena	trnT	9482	9547	66	J	0			UGU
A. xanthoabdomena	trnP	9548	9615	68	N	2			UGG
A. xanthoabdomena	ND6	9618	10098	481	J	0	ATT	T	
A. xanthoabdomena	CYTB	10,099	11,235	1137	J	-2	ATG	TAG	
A. xanthoabdomena	trnS2	11,234	11,298	65	J	-1			UGA
A. xanthoabdomena	ND1	11,298	12,230	933	N	0	ATT	TAA	
A. xanthoabdomena	trnL2	12,231	12,293	63	N	0			UAG
A. xanthoabdomena	l-rRNA	12,294	13,489	1196	N	0			
A. xanthoabdomena	trnV	13,490	13,553	64	N	0			UAC
A. xanthoabdomena	s-rRNA	13,554	14,283	730	N	0			
A. xanthoabdomena	Control region	14,284	15,463	1180					
A. yunnanana	trnI	1	64	64	J	-3			GAU
A. yunnanana	trnQ	62	129	68	N	5			UUG
A. yunnanana	trnM	135	203	69	J	0			CAU
A. yunnanana	ND2	204	1175	972	J	-2	ATT	TAA	
A. yunnanana	trnW	1174	1238	65	J	-8			UCA
A. yunnanana	trnC	1231	1293	63	N	3			GCA
A. yunnanana	trnY	1297	1360	64	N	4			GUA
A. yunnanana	COX1	1365	2900	1536	J	0	ATG	TAG	
A. yunnanana	trnL1	2901	2965	65	J	0			UAA
A. yunnanana	COX2	2966	3644	679	J	0	ATT	T	

A. yunnanana	trnK	3645	3716	72	J	-1			CUU
A. yunnanana	trnD	3716	3780	65	J	0			GUC
A. yunnanana	ATP8	3781	3933	153	J	-7	TTG	TAA	
A. yunnanana	ATP6	3927	4580	654	J	0	ATG	TAA	
A. yunnanana	COX3	4581	5359	779	J	-1	ATG	TA	
A. yunnanana	trnG	5359	5422	64	J	0			UCC
A. yunnanana	ND3	5423	5776	354	J	3	ATT	TAA	
A. yunnanana	trnA	5780	5841	62	J	-2			UGC
A. yunnanana	trnR	5840	5906	67	J	-2			UCG
A. yunnanana	trnN	5905	5973	69	J	-1			GUU
A. yunnanana	trnS1	5973	6038	66	J	1			GCU
A. yunnanana	trnE	6040	6105	66	J	-1			UUC
A. yunnanana	trnF	6105	6169	65	N	0			GAA
A. yunnanana	ND5	6170	7844	1675	N	0	TTG	T	
A. yunnanana	trnH	7845	7905	61	N	-1			GUG
A. yunnanana	ND4	7905	9227	1323	N	-7	ATG	TAA	
A. yunnanana	ND4L	9221	9502	282	N	2	ATG	TAA	
A. yunnanana	trnT	9505	9568	64	J	0			UGU
A. yunnanana	trnP	9569	9636	68	N	2			UGG
A. yunnanana	ND6	9639	10,119	481	J	0	ATT	T	
A. yunnanana	CYTB	10,120	11,256	1137	J	-2	ATG	TAG	
A. yunnanana	trnS2	11,255	11,320	66	J	-1			UGA
A. yunnanana	ND1	11,320	12,252	933	N	0	ATT	TAA	
A. yunnanana	trnL2	12,253	12,315	63	N	0			UAG
A. yunnanana	l-rRNA	12,316	13,509	1194	N	0			
A. yunnanana	trnV	13,510	13,574	65	N	0			UAC

A. yunnanana	s-rRNA	13,575	14,307	733	N	0
A. yunnanana	Control region	14,308	15,875	1568		
