# Supplementary material for: Comparative Analysis of Mitochondrial Genomes among Twelve Sibling Species of the Genus Atkinsoniella Distant, 1908 (Hemiptera: Cicadellidae: Cicadellinae) and Phylogenetic Analysis
Source: Insects. 2022 Mar 3;13(3):254. doi: 10.3390/insects13030254 (PMC8953490; doi:10.3390/insects13030254)
Supplement: Supplementary file 1 [file insects-13-00254-s001.zip › Table S3.pdf]

Table S3. Nucleotide composition of the 12 newly sequenced *Atkinsoniella* mitogenomes.

Species	Regions	Length(bp)	T%	C%	A%	G%	A+T%	AT skew	GC skew
A. aurantiaca	Whole genome	15,394	37.0	11.0	42.2	9.8	79.2	0.066	-0.055
	PCGs	10,953	44.7	10.6	33.2	11.6	77.9	-0.148	0.044
	1st codon position	3651	37.1	10.4	36.7	15.8	73.8	-0.006	0.209
	2nd codon position	3651	48.6	16.8	20.7	13.9	69.3	-0.404	-0.095
	3rd codon position	3651	48.3	4.6	42.1	5.0	90.4	-0.068	0.037
	tRNAs	1434	39.6	8.5	40.9	10.9	80.5	0.016	0.125
	rRNAs	1933	47.9	7.3	33.0	11.8	80.9	-0.185	0.238
	Control region	1095	44.9	5.7	43.2	6.2	88.1	-0.020	0.046
A. curvata	Whole genome	15,988	36.5	11.4	42.8	9.3	79.3	0.079	-0.098
	PCGs	10,953	44.3	10.8	33.2	11.7	77.5	-0.144	0.039
	1st codon position	3651	36.8	10.5	36.6	16.0	73.5	-0.003	0.205
	2nd codon position	3651	48.7	16.8	20.6	13.9	69.3	-0.406	-0.097
	3rd codon position	3651	47.5	5.0	42.3	5.2	89.8	-0.058	0.016
	tRNAs	1439	39.4	8.5	41.1	11.0	80.5	0.021	0.125
	rRNAs	1929	48.2	7.1	32.9	11.9	81.1	-0.188	0.255
	Control region	1687	43.2	6.5	44.9	5.4	88.1	0.020	-0.095
A. flavipenna	Whole genome	15,671	36.7	11.4	42.5	9.5	79.1	0.073	-0.093
	PCGs	10,953	44.4	11.0	33.2	11.4	77.6	-0.144	0.017
	1st codon position	3651	37.1	10.4	37.0	15.5	74.1	-0.001	0.197
	2nd codon position	3651	48.4	17.1	20.7	13.8	69.1	-0.400	-0.105
	3rd codon position	3651	47.6	5.6	41.9	4.9	89.5	-0.064	-0.070
	tRNAs	1443	39.4	8.3	41.2	11.1	80.6	0.021	0.143
	rRNAs	1927	47.5	7.2	33.8	11.5	81.4	-0.168	0.231

	Control region	1368	44.0	7.7	42.6	5.7	86.6	-0.016	-0.148
A. longiuscula	Whole genome	15,815	37.0	11.3	42.3	9.4	79.2	0.067	-0.092
	PCGs	10,953	44.3	11.1	33.3	11.3	77.7	-0.142	0.010
	1st codon position	3651	37.1	10.4	36.9	15.6	74.0	-0.002	0.202
	2nd codon position	3651	48.4	17.0	20.8	13.8	69.1	-0.399	-0.104
	3rd codon position	3651	47.6	5.8	42.3	4.4	89.9	-0.059	-0.138
	tRNAs	1443	39.6	8.2	41.0	11.2	80.5	0.017	0.153
	rRNAs	1929	47.7	7.2	33.6	11.5	81.3	-0.173	0.233
	Control region	1510	45.7	7.3	41.0	6.0	86.7	-0.054	-0.095
A. thalia	Whole genome	15,034	35.0	12.3	42.7	10.0	77.8	0.098	-0.103
	PCGs	10,953	43.8	11.5	32.6	12.1	76.4	-0.146	0.025
	1st codon position	3651	36.3	11.2	36.2	16.4	72.4	-0.001	0.189
	2nd codon position	3651	48.4	16.8	20.7	14.1	69.1	-0.402	-0.086
	3rd codon position	3651	46.6	6.6	41.1	5.8	87.6	-0.063	-0.064
	tRNAs	1436	39.5	8.8	40.5	11.3	79.9	0.012	0.125
	rRNAs	1922	49.0	7.5	31.7	11.8	80.7	-0.213	0.224
	Control region	744	37.8	8.2	48.3	5.8	86.0	0.122	-0.173
A. thaloidea	Whole genome	15,571	35.0	12.5	43.2	9.4	78.2	0.104	-0.141
	PCGs	10,953	43.8	11.6	32.8	11.9	76.5	-0.144	0.012
	1st codon position	3651	36.4	11.2	36.2	16.1	72.6	-0.002	0.179
	2nd codon position	3651	48.4	16.8	20.7	14.1	69.1	-0.400	-0.086
	3rd codon position	3651	46.5	6.8	41.3	5.4	87.8	-0.059	-0.115
	tRNAs	1435	39.9	8.7	40.4	11.0	80.3	0.007	0.117
	rRNAs	1923	49.8	7.0	31.4	11.8	81.2	-0.227	0.258
	Control region	1282	41.3	8.8	44.1	5.7	85.5	0.033	-0.215

A. tiani	Whole genome	15,841	36.2	11.6	43.0	9.3	79.2	0.085	-0.110
	PCGs	10,953	44.2	11.1	33.2	11.5	77.4	-0.142	0.020
	1st codon position	3651	36.8	10.6	36.8	15.8	73.6	-0.001	0.199
	2nd codon position	3651	48.5	16.8	20.8	14.0	69.3	-0.400	-0.091
	3rd codon position	3651	47.2	5.9	42.0	4.8	89.2	-0.058	-0.104
	tRNAs	1436	40.1	8.2	40.3	11.4	80.4	0.003	0.160
	rRNAs	1920	49.0	7.2	32.1	11.8	81.0	-0.208	0.242
	Control region	1552	43.0	7.9	45.6	3.5	88.6	0.030	-0.379
A. uniguttata	Whole genome	15,784	36.7	11.1	43.1	9.1	79.8	0.080	-0.098
	PCGs	10,953	44.5	10.6	33.9	11.0	78.4	-0.136	0.019
	1st codon position	3651	37.2	10.3	36.9	15.7	74.0	-0.004	0.207
	2nd codon position	3651	48.6	16.7	20.9	13.8	69.5	-0.398	-0.093
	3rd codon position	3651	47.9	4.8	43.8	3.5	91.7	-0.045	-0.158
	tRNAs	1432	40.0	8.0	41.3	10.6	81.4	0.016	0.139
	rRNAs	1919	48.2	7.1	33.3	11.4	81.4	-0.182	0.230
	Control region	1492	42.4	7.4	44.0	6.1	86.5	0.019	-0.099
A. warpa	Whole genome	15,852	35.5	11.7	43.5	9.2	79.0	0.101	-0.118
	PCGs	10,953	44.1	11.0	33.2	11.6	77.4	-0.141	0.027
	1st codon position	3651	36.7	10.6	36.6	16.0	73.3	-0.001	0.202
	2nd codon position	3651	48.4	16.8	20.8	14.0	69.2	-0.398	-0.091
	3rd codon position	3651	47.3	5.6	42.2	4.8	89.5	-0.057	-0.073
	tRNAs	1436	40.2	8.1	40.5	11.2	80.6	0.003	0.158
	rRNAs	1919	49.1	7.2	32.0	11.6	81.1	-0.211	0.232
	Control region	1562	37.1	9.3	49.6	4.0	86.6	0.144	-0.397
A. wui	Whole genome	15,159	35.2	12.3	42.5	10.0	77.7	0.094	-0.103

	PCGs	10,953	43.4	11.4	33.0	12.1	76.4	-0.136	0.030
	1st codon position	3651	36.4	10.9	36.5	16.2	72.9	0.002	0.195
	2nd codon position	3651	48.5	16.7	20.9	14.0	69.4	-0.398	-0.088
	3rd codon position	3651	45.4	6.7	41.7	6.2	87.0	-0.042	-0.036
	tRNAs	1426	39.7	8.3	40.5	11.6	80.2	0.010	0.166
	rRNAs	1922	48.9	7.2	32.0	11.8	81.0	-0.208	0.240
	Control region	883	40.1	8.5	43.6	7.8	83.7	0.042	-0.042
A. xanthoabdomena	Whole genome	15,463	36.3	11.7	42.5	9.4	78.9	0.078	-0.108
	PCGs	10,953	43.8	11.2	33.6	11.5	77.4	-0.132	0.015
	1st codon position	3651	36.7	10.6	36.6	16.1	73.3	-0.001	0.207
	2nd codon position	3651	48.3	16.8	21.0	13.9	69.3	-0.393	-0.096
	3rd codon position	3651	46.3	6.1	43.1	4.5	89.5	-0.036	-0.148
	tRNAs	1429	39.4	8.7	40.9	10.9	80.3	0.019	0.110
	rRNAs	1926	46.3	7.4	34.7	11.6	81.0	-0.142	0.219
	Control region	1180	43.6	6.3	44.2	5.8	87.9	0.007	-0.035
A. yunnanana	Whole genome	15,875	36.2	11.5	42.7	9.5	79.0	0.082	-0.093
	PCGs	10,953	44.3	10.7	33.5	11.5	77.8	-0.140	0.035
	1st codon position	3651	36.2	10.8	36.8	16.2	73.0	0.009	0.201
	2nd codon position	3651	48.5	16.7	20.8	14.0	69.3	-0.399	-0.090
	3rd codon position	3651	48.3	4.6	42.8	4.3	91.1	-0.061	-0.040
	tRNAs	1441	40.2	8.2	40.8	10.8	81.0	0.008	0.139
	rRNAs	1927	48.2	7.1	33.2	11.6	81.4	-0.185	0.242
	Control region	1568	40.2	9.4	41.8	8.5	82.1	0.019	-0.053
