# Supplementary material for: Comparative Analysis of Mitochondrial Genomes among Twelve Sibling Species of the Genus Atkinsoniella Distant, 1908 (Hemiptera: Cicadellidae: Cicadellinae) and Phylogenetic Analysis
Source: Insects. 2022 Mar 3;13(3):254. doi: 10.3390/insects13030254 (PMC8953490; doi:10.3390/insects13030254)
Supplement: Supplementary file 1 [file insects-13-00254-s001.zip › Table S4.pdf]

Table S4. The start codons and stop codons of each protein coding gene in the 12 *Atkinsoniella* mitogenomes

**Gene**	**Start Codon/Stop Codon**											
	A. aurantiaca	***A. curvata***	***A. flavipenna***	***A. longiuscula***	***A. thalia***	***A. thaloidea***	***A. tiani***	***A. uniguttata***	***A. warpa***	***A. wui***	***A. xanthoabdomena***	***A. yunnanana***
ND2	ATT/TAA	ATT/TAA	ATT/TAA	ATT/TAA	ATT/TAA	ATT/TAA	ATT/TAA	ATA/TAA	ATT/TAA	ATT/TAA	ATT/TAA	ATT/TAA
COX1	ATG/TAA	ATG/TAA	ATG/TAA	ATG/TAA	ATG/TAA	ATG/TAA	ATG/TAA	ATG/TAA	ATG/TAA	ATG/TAA	ATG/TAA	ATG/TAG
COX2	ATT/T	ATT/T	ATT/T	ATT/T	ATT/T	ATT/T	ATT/T	ATT/T	ATT/T	ATT/T	ATT/T	ATT/T
ATP8	TTG/TAA	TTG/TAA	TTG/TAG	TTG/TAG	TTG/TAA	TTG/TAA	TTG/TAA	TTG/TAA	TTG/TAA	TTG/TAA	TTG/TAA	TTG/TAA
ATP6	GTG/TAA	ATG/TAA	GTG/TAA	GTG/TAA	ATG/TAA	ATG/TAA	ATG/TAA	ATG/TAA	ATG/TAA	ATG/TAA	ATG/TAA	ATG/TAA
COX3	ATG/TA	ATG/TA	ATG/TA	ATG/TA	ATG/TA	ATG/TA	ATG/TA	ATG/TA	ATG/TA	ATG/TA	ATG/TA	ATG/TA
ND3	ATT/TAA	ATT/TAA	ATC/TAA	ATC/TAA	ATC/TAA	ATC/TAA	ATT/TAA	ATA/TAA	ATT/TAA	ATC/TAA	ATT/TAA	ATT/TAA
ND5	TTG/T	TTG/T	TTG/T	TTG/T	TTG/T	TTG/T	TTG/T	TTG/T	TTG/T	TTG/T	TTG/T	TTG/T
ND4	ATG/TAA	ATG/TAA	ATG/TAA	ATG/TAA	ATG/TAA	ATG/TAA	ATG/TAA	ATG/TAA	ATG/TAA	ATG/TAA	ATG/TAA	ATG/TAA
ND4L	ATG/TAA	ATG/TAA	ATG/TAA	ATG/TAA	ATG/TAA	ATG/TAA	ATG/TAA	ATG/TAA	ATG/TAA	ATG/TAA	ATG/TAA	ATG/TAA
ND6	ATT/T	ATT/T	ATT/T	ATT/T	ATT/T	ATT/T	ATT/T	ATT/T	ATT/T	ATT/T	ATT/T	ATT/T
CYTB	ATG/TAG	ATG/TAG	ATG/TAG	ATG/TAG	ATG/TAG	ATG/TAG	ATG/TAG	ATG/TAG	ATG/TAG	ATG/TAG	ATG/TAG	ATG/TAG
ND1	ATT/TAA	ATT/TAA	ATT/TAA	ATT/TAA	ATT/TAA	ATT/TAA	ATT/TAA	ATT/TAA	ATT/TAA	ATT/TAA	ATT/TAA	ATT/TAA

The codons on the left and right of slashes (/) are the start codons and stop codons, respectively.
