# Supplementary material for: Comparative Analysis of Mitochondrial Genomes among Twelve Sibling Species of the Genus Atkinsoniella Distant, 1908 (Hemiptera: Cicadellidae: Cicadellinae) and Phylogenetic Analysis
Source: Insects. 2022 Mar 3;13(3):254. doi: 10.3390/insects13030254 (PMC8953490; doi:10.3390/insects13030254)
Supplement: Supplementary file 1 [file insects-13-00254-s001.zip › Table S6.pdf]

Table S6. Genetic distances for the 14 *Atkinsoniella* species in this study.

speceis	1	2	3	4	5	6	7	8	9	10	11	12	13	14
A. aurantiaca		0.003	0.003	0.003	0.004	0.004	0.004	0.004	0.004	0.004	0.004	0.004	0.005	0.005
A. curvata	0.077		0.003	0.003	0.004	0.004	0.004	0.004	0.004	0.004	0.004	0.004	0.005	0.005
A. flavipenna	0.091	0.091		0.002	0.004	0.004	0.004	0.004	0.004	0.004	0.004	0.004	0.005	0.005
A. longiuscula	0.092	0.089	0.035		0.004	0.004	0.004	0.004	0.004	0.004	0.004	0.004	0.005	0.005
A. thalia	0.135	0.130	0.131	0.133		0.003	0.003	0.004	0.003	0.004	0.004	0.004	0.005	0.005
A. thaloidea	0.133	0.131	0.131	0.131	0.076		0.003	0.004	0.003	0.004	0.004	0.004	0.005	0.005
A. tiani	0.127	0.127	0.121	0.120	0.104	0.104		0.004	0.002	0.004	0.004	0.004	0.005	0.005
A. uniguttata	0.134	0.134	0.132	0.134	0.142	0.146	0.139		0.004	0.004	0.004	0.004	0.005	0.005
A. warpa	0.127	0.125	0.122	0.121	0.103	0.102	0.026	0.139		0.004	0.004	0.004	0.005	0.005
A. wui	0.142	0.141	0.142	0.139	0.135	0.136	0.129	0.150	0.127		0.004	0.004	0.005	0.005
A. xanthoabdomena	0.146	0.144	0.142	0.142	0.152	0.153	0.147	0.148	0.147	0.161		0.004	0.005	0.005
A. yunnanana	0.122	0.124	0.119	0.120	0.133	0.134	0.122	0.136	0.122	0.140	0.143		0.005	0.005
A. grahami	0.202	0.202	0.202	0.201	0.210	0.211	0.202	0.201	0.202	0.212	0.211	0.200		0.002
A. xanthonota	0.203	0.203	0.203	0.204	0.214	0.213	0.204	0.203	0.204	0.214	0.213	0.203	0.037	

Note: Interspecific distances were calculated using the pairwise distance model in MEGA 6.0.
