# Supplementary material for: Comparative Analysis of Mitochondrial Genomes among Twelve Sibling Species of the Genus Atkinsoniella Distant, 1908 (Hemiptera: Cicadellidae: Cicadellinae) and Phylogenetic Analysis
Source: Insects. 2022 Mar 3;13(3):254. doi: 10.3390/insects13030254 (PMC8953490; doi:10.3390/insects13030254)
Supplement: Supplementary file 1 [file insects-13-00254-s001.zip › Table S7.pdf]

**Table S7.** Best models were calculated by Modelfinder of cds_faa, cds12_fna, cds12_rrna datasets used in analysis

| Dataset | Subset | SubsetPartitions | BestModel |
|---|---|---|---|
| cds_faa | P1 | COX1_aa | mtART+I+G4 |
| | P2 | ND1_aa_ND4_aa_ND5_aa_ND4L_aa | mtZOA+F+R5 |
| | P3 | COX2_aa_ND2_aa_COX3_aa_ND3_aa_ATP6_aa_ND6_aa_ATP8_aa_CYTB_aa | mtART+F+R5 |
| cds12_fna | P1 | ATP6.nuc_ATP8.nuc_ND2.nuc_ND3.nuc_ND6.nuc | GTR+F+I+G4 |
| | P2 | COX1.nuc_CYTB.nuc | GTR+F+R3 |
| | P3 | COX2.nuc_COX3.nuc | GTR+F+I+G4 |
| | P4 | ND1.nuc_ND4.nuc_ND4L.nuc_ND5.nuc | TVM+F+R4 |
| cds12_rrna | P1 | ATP6.nuc_COX2.nuc_COX3.nuc | TIM+F+I+G4 |
| | P2 | ATP8.nuc_ND3.nuc_ND6.nuc | TIM+F+G4 |
| | P3 | COX1.nuc_CYTB.nuc | GTR+F+I+G4 |
| | P4 | ND1.nuc_ND4.nuc_ND4L.nuc_ND5.nuc | TVM+F+I+G4 |
| | P5 | ND2.nuc | TVM+F+I+G4 |
| | P6 | 12s.nuc_16s.nuc | TVM+F+G4 |
