# Supplementary material for: Comparative Analysis of Mitochondrial Genomes among Twelve Sibling Species of the Genus Atkinsoniella Distant, 1908 (Hemiptera: Cicadellidae: Cicadellinae) and Phylogenetic Analysis
Source: Insects. 2022 Mar 3;13(3):254. doi: 10.3390/insects13030254 (PMC8953490; doi:10.3390/insects13030254)
Supplement: Supplementary file 1 [file insects-13-00254-s001.zip › Table S8.pdf]

Table S8. Best models were calculated by PartitionFinder2 of cds_faa, cds12_fna, cds12_rrna datasets used in analysis

**Dataset**	**Subset**	**Subset Partitions**	**Best Model**
cds_faa	P1	COX1_aa	MTREV+I+G
	P2	ND1_aa	MTMAM+I+G
	P1	CYTB_aa, COX3_aa, COX2_aa	MTREV+I+G
	P2	ND2_aa, ATP8_aa	MTREV+I+G
	P3	ND4L_aa, ND3_aa	MTREV+G
	P4	ND4_aa, ND6_aa	MTREV+I+G
	P5	ND5_aa, ATP6_aa	MTREV+I+G
cds12_fna	P1	COX1_nuc	RTREV+I+G
	P2	ND1_nuc	DAYHOFF+I+G
	P3	CYTB_nuc, COX2_nuc, COX3_nuc	RTREV+I+G
	P4	ND2_nuc, ATP8_nuc	RTREV+I+G
	P5	ATP6_nuc, ND6_nuc, ND3_nuc	RTREV+I+G
	P6	ND4_nuc, ND5_nuc, ND4L_nuc	DAYHOFF+I+G
cds12_rrna	P1	ND1_nuc, ND4_nuc, ND4L_nuc, ND5_nuc	GTR+I+G
	P2	COX2_nuc, COX3_nuc, CYTB_nuc, COX1_nuc	GTR+I+G
	P3	ND2_nuc	GTR+I+G
	P4	ATP8_nuc, ND3_nuc, ND6_nuc, ATP6_nuc	GTR+I+G
	P5	12s, 16s	GTR+G
