# Supplementary figures and images for: Comparative Analysis of Mitochondrial Genomes among Twelve Sibling Species of the Genus Atkinsoniella Distant, 1908 (Hemiptera: Cicadellidae: Cicadellinae) and Phylogenetic Analysis
Source: Insects. 2022 Mar 3;13(3):254. doi: 10.3390/insects13030254 (PMC8953490; doi:10.3390/insects13030254)

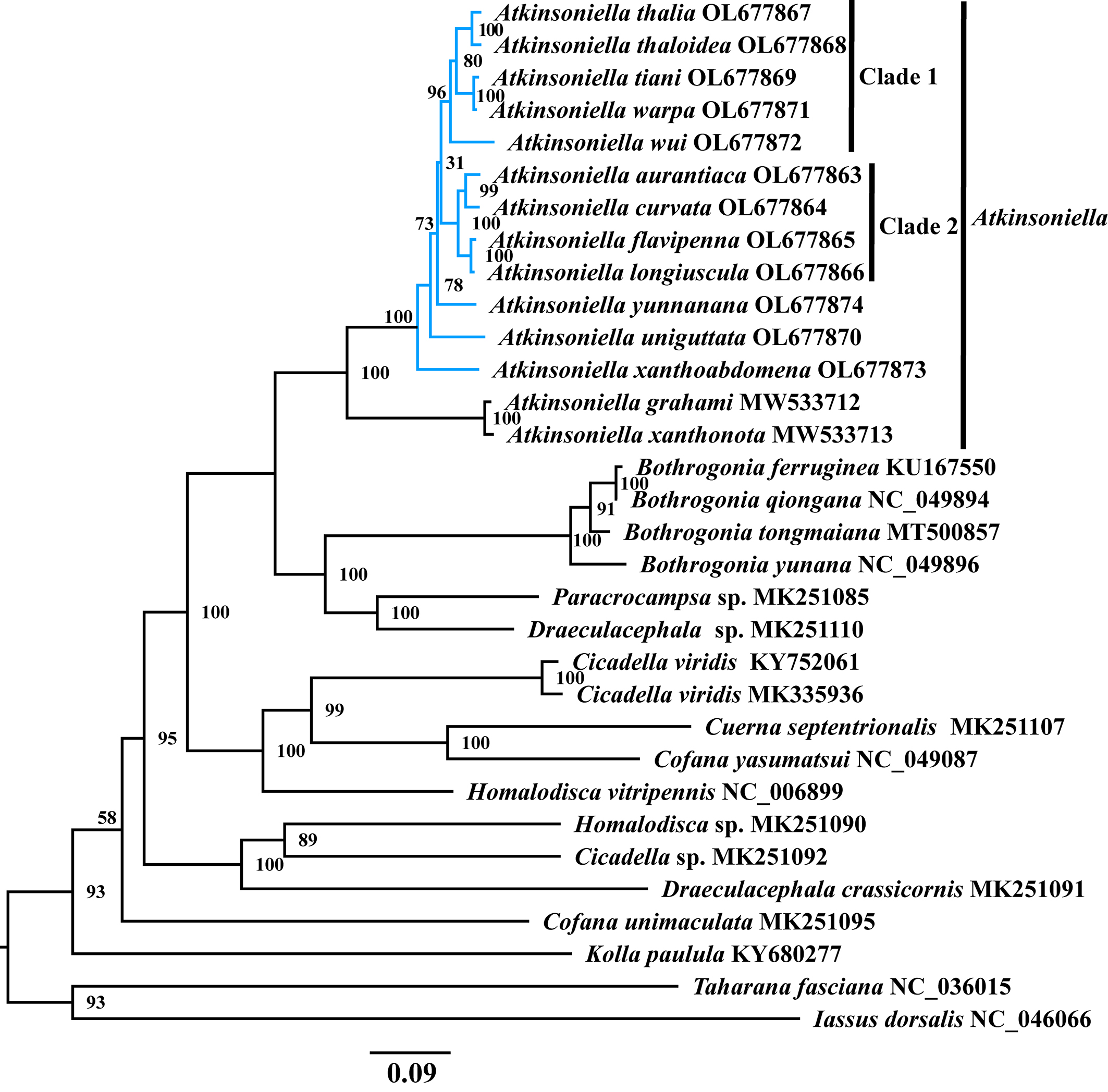

Supplement: Supplementary file 1 [file insects-13-00254-s001.zip › Fig. S1.jpg]

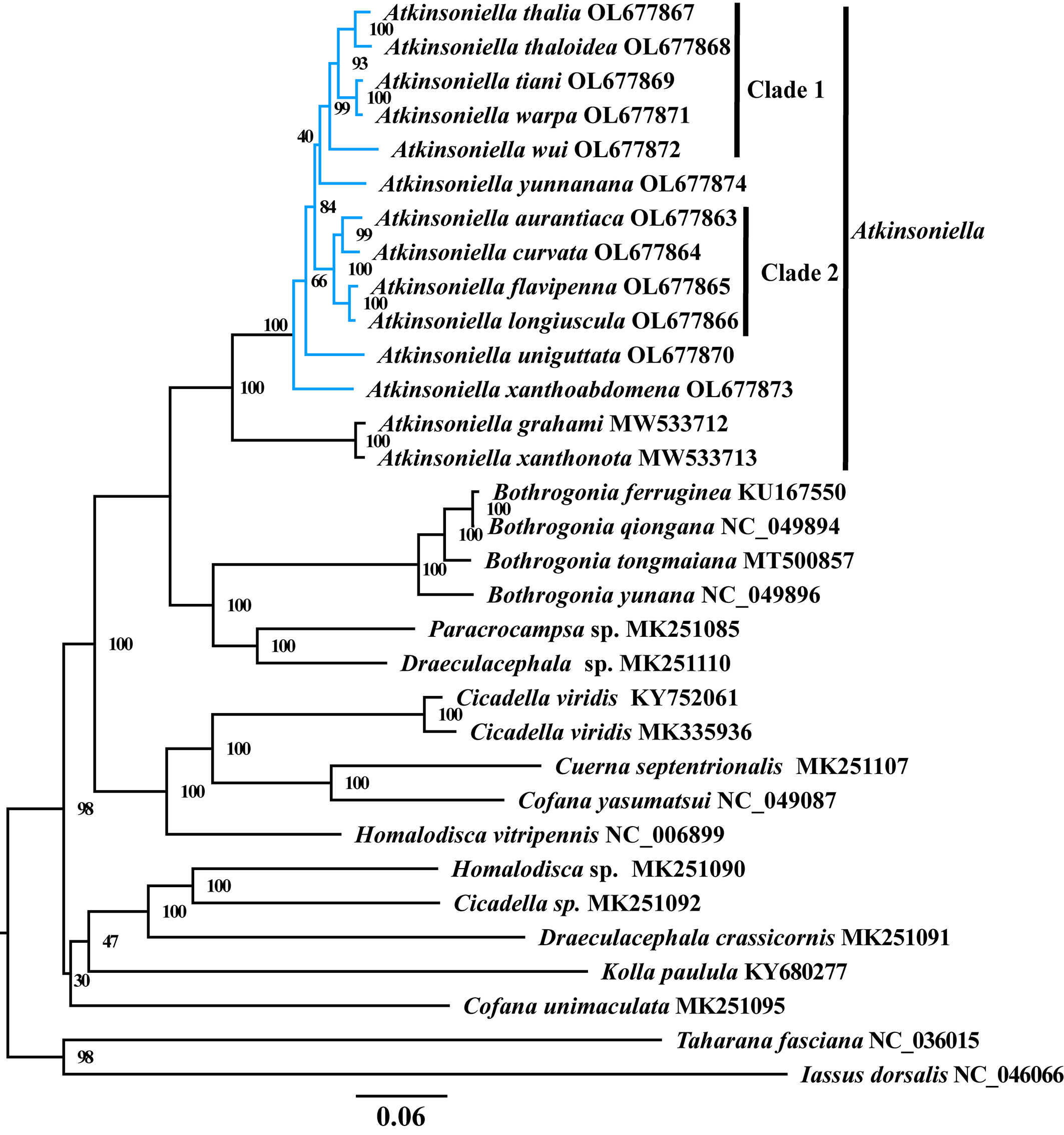

Supplement: Supplementary file 1 [file insects-13-00254-s001.zip › Fig. S2.jpg]

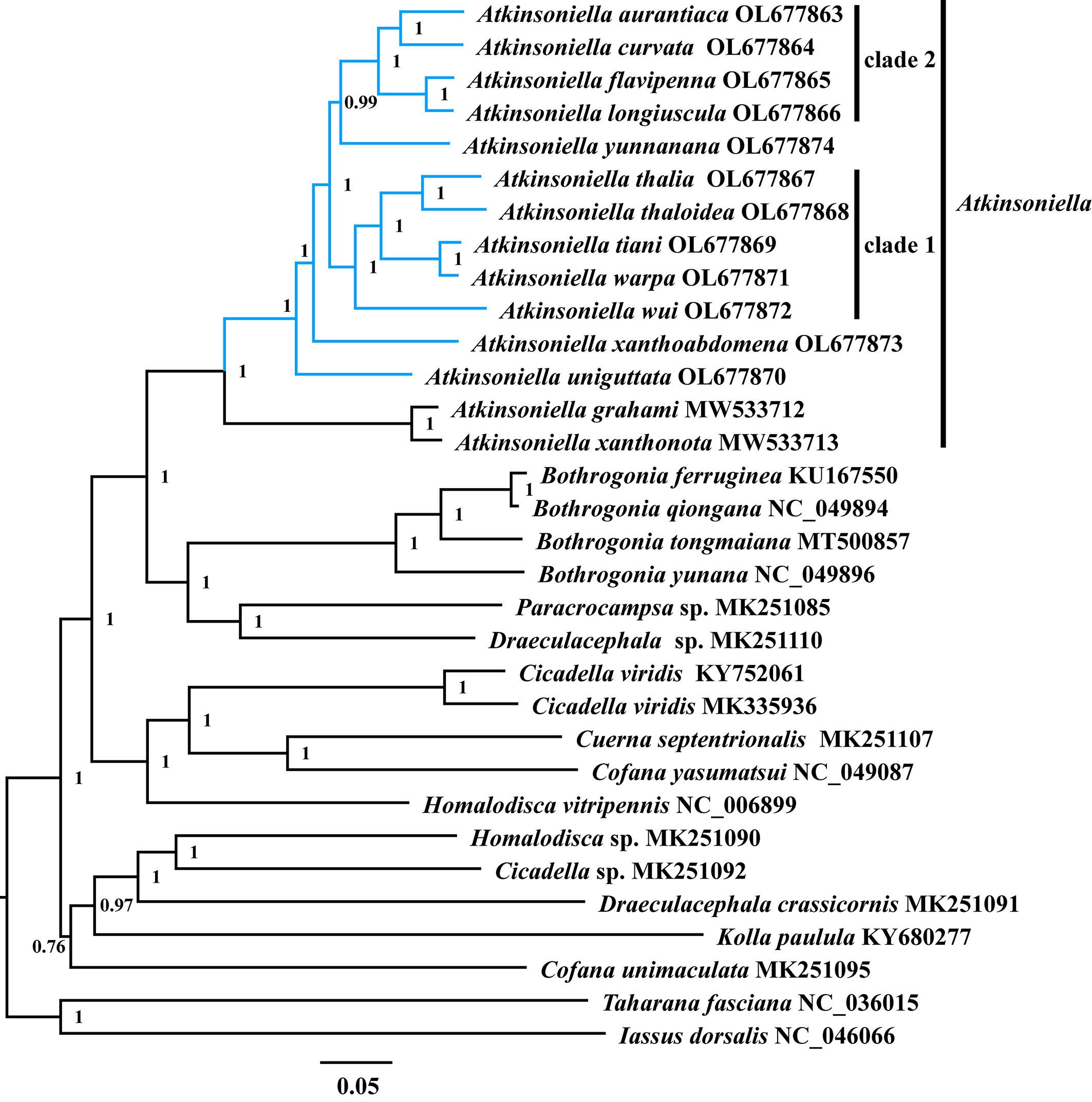

Supplement: Supplementary file 1 [file insects-13-00254-s001.zip › Fig. S3.jpg]

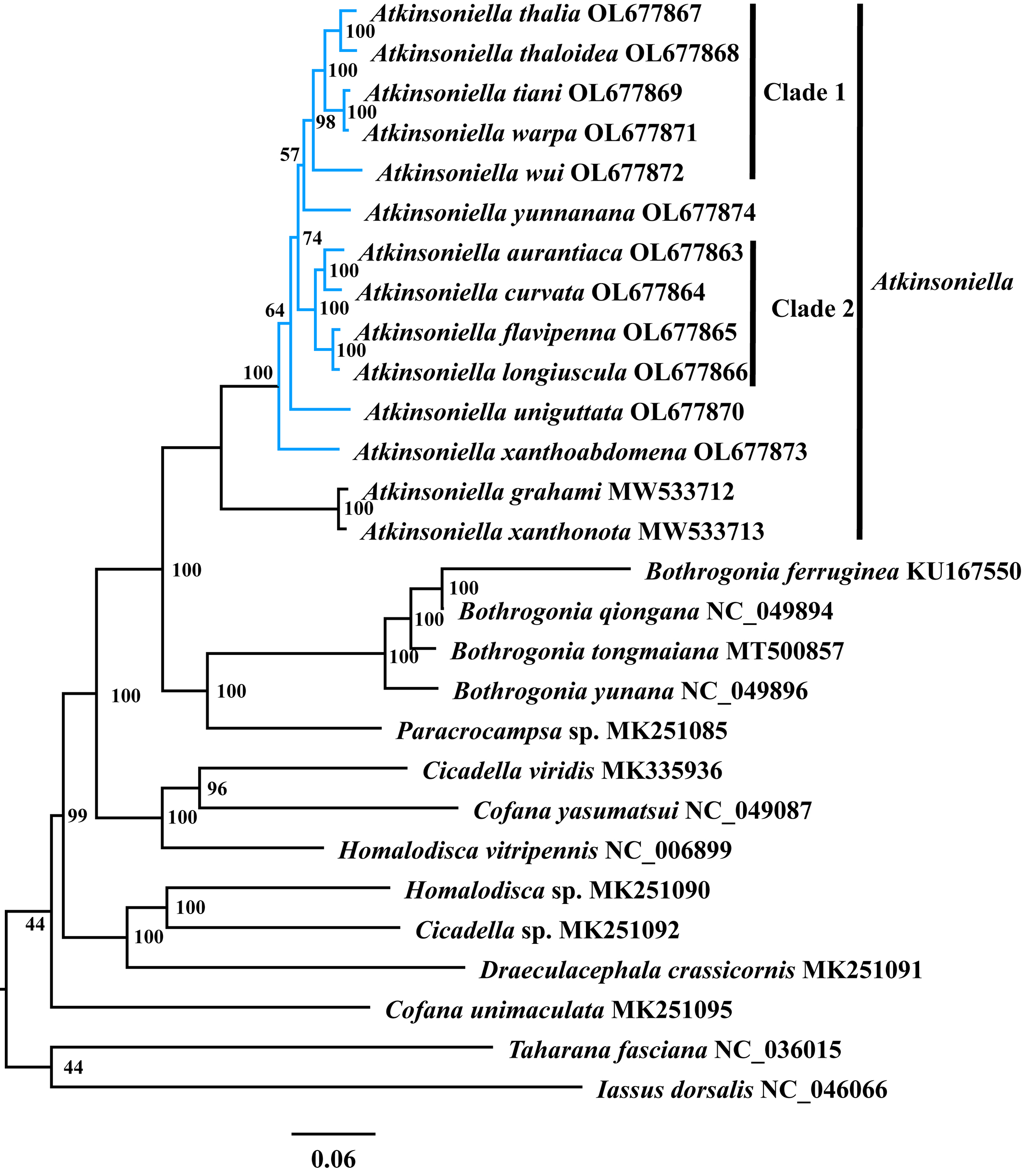

Supplement: Supplementary file 1 [file insects-13-00254-s001.zip › Fig. S4.jpg]

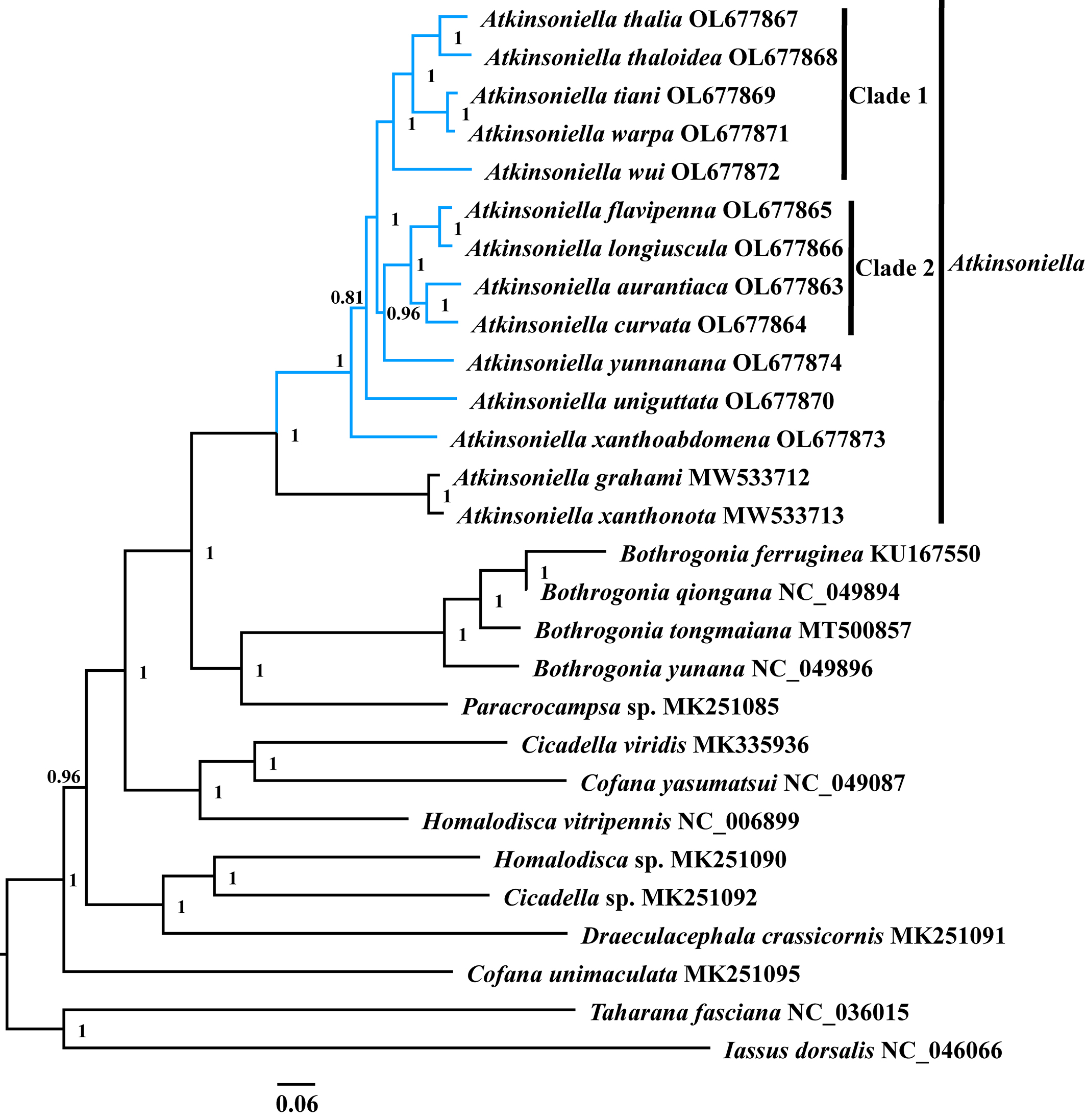

Supplement: Supplementary file 1 [file insects-13-00254-s001.zip › Fig. S5.jpg]
